# Supplementary figures and images for: Senescence in yeast is associated with amplified linear fragments of chromosome XII rather than ribosomal DNA circle accumulation
Source: PLoS Biol. 2023 Aug 29;21(8):e3002250. doi: 10.1371/journal.pbio.3002250 (PMC10464983; doi:10.1371/journal.pbio.3002250)

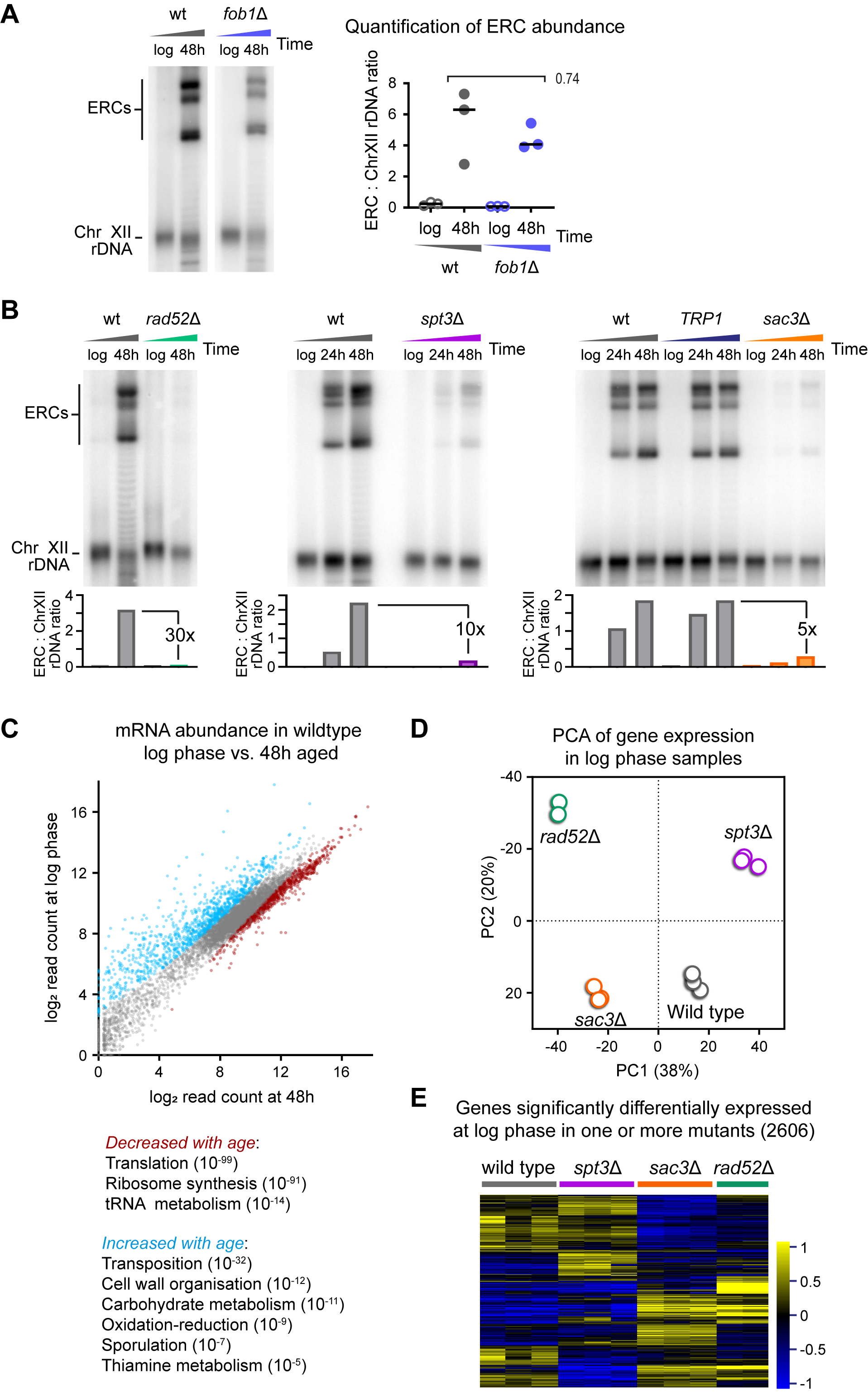

Supplement: S1 Fig — (A) Southern blot analysis of ERC abundance in log phase and 48-hour-aged wild-type and fob1Δ MEP cells. Quantification shows ratio between sum of ERC bands and chromosome XII band for 3 biological replicates, p-value calculated by 1-way ANOVA. Images show 2 sections of the same blot membrane with no differential image processing applied. (B) Southern blot analysis of ERC abundance at log phase and indicated aged time points for wild type, rad52Δ, spt3Δ, and sac3Δ mutant MEP cells, along with a wild type in which the TRP1 gene has been restored. (C) Scatter plot of log2 mRNA abundance comparing log phase to 48-hour-aged wild type. Sets of genes called as significantly differentially expressed by DEseq2 (BH-corrected FDR < 0.05, log2-fold change threshold ±0.5) are highlighted in colour. GO terms are provided for each category, significance indicated by FDR-corrected q values, full GO term enrichment analysis in S7 File. (C) PCA summary for log phase poly(A)+ RNA-seq libraries from wild type and rad52Δ, spt3Δ, and sac3Δ mutants. (E) Hierarchical clustering analysis of the 2,606 genes significantly differentially expressed at log phase between at least 1 mutant and the wild type by DEseq2 (BH-corrected FDR < 0.01, log2-fold change threshold ±0.5). Individual biological replicates are shown; note that scale is different to other hierarchical clustering analyses presented as differences between log phase samples are smaller than age-linked changes. The numerical data underlying this Figure can be found in S8 File. BH, Benjamini–Hochberg; ERC, extrachromosomal ribosomal DNA circle; FDR, false discovery rate; GO, gene ontology; MEP, mother enrichment program; PCA, principal component analysis. (TIF) [file pbio.3002250.s001.tif]

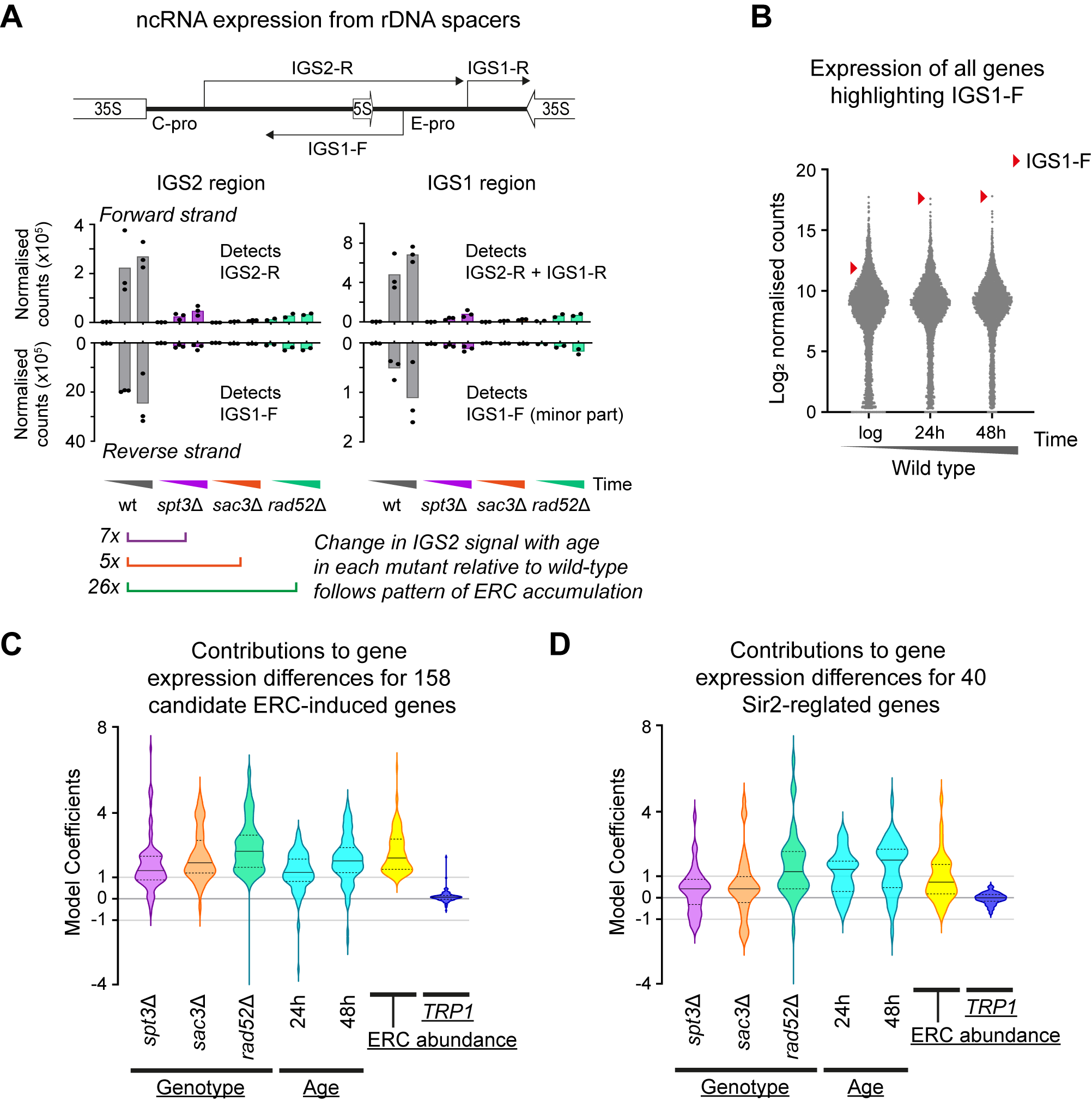

Supplement: S2 Fig — (A) Schematic of known RNA polymerase II transcripts in the intergenic spacer region of the rDNA, and quantification of noncoding transcripts orientated sense and antisense to the RNA polymerase I–transcribed 35S genes. Individual biological replicates are shown as points (3 for wild type, spt3Δ, and sac3Δ and 2 for rad52Δ), and bars show mean values for a given age and genotype. (B) Distribution of log2 mean normalised mRNA abundance for all genes in wild-type cells, including probes to rDNA intergenic spacer regions. Red arrows highlight the probe that detects the IGS1-F noncoding RNA as defined in panel A. (C) Coefficients representing the contribution of each individual DESeq2 linear model factor to mean log2 normalised mRNA abundance of cluster 2 genes (from Fig 2B) across all datasets. DESeq2 was used to model expression as dependent on 4 categorical independent variables (with an additional intercept factor): Genotype (wild type, spt3Δ, rad52Δ, sac3Δ), TRP1 auxotrophy (present, not present), Age (log phase, 24 hours, 48 hours), ERC accumulation (low, high). Coefficient values can be interpreted as the log2 normalised mRNA abundance change caused by a specific model factor being present relative to a wild-type log phase TRP1 auxotroph with low ERC levels. Solid horizontal lines within violins indicate median values, while lower and upper quartiles are indicated by dotted horizontal lines. Full output of the model is provided in S1 File. (D) As for C showing values for the 40 SIR-complex regulated genes (from Ellahi and colleagues) across all datasets. The numerical data underlying this Figure can be found in S8 File. ERC, extrachromosomal ribosomal DNA circle; rDNA, ribosomal DNA. (TIF) [file pbio.3002250.s002.tif]

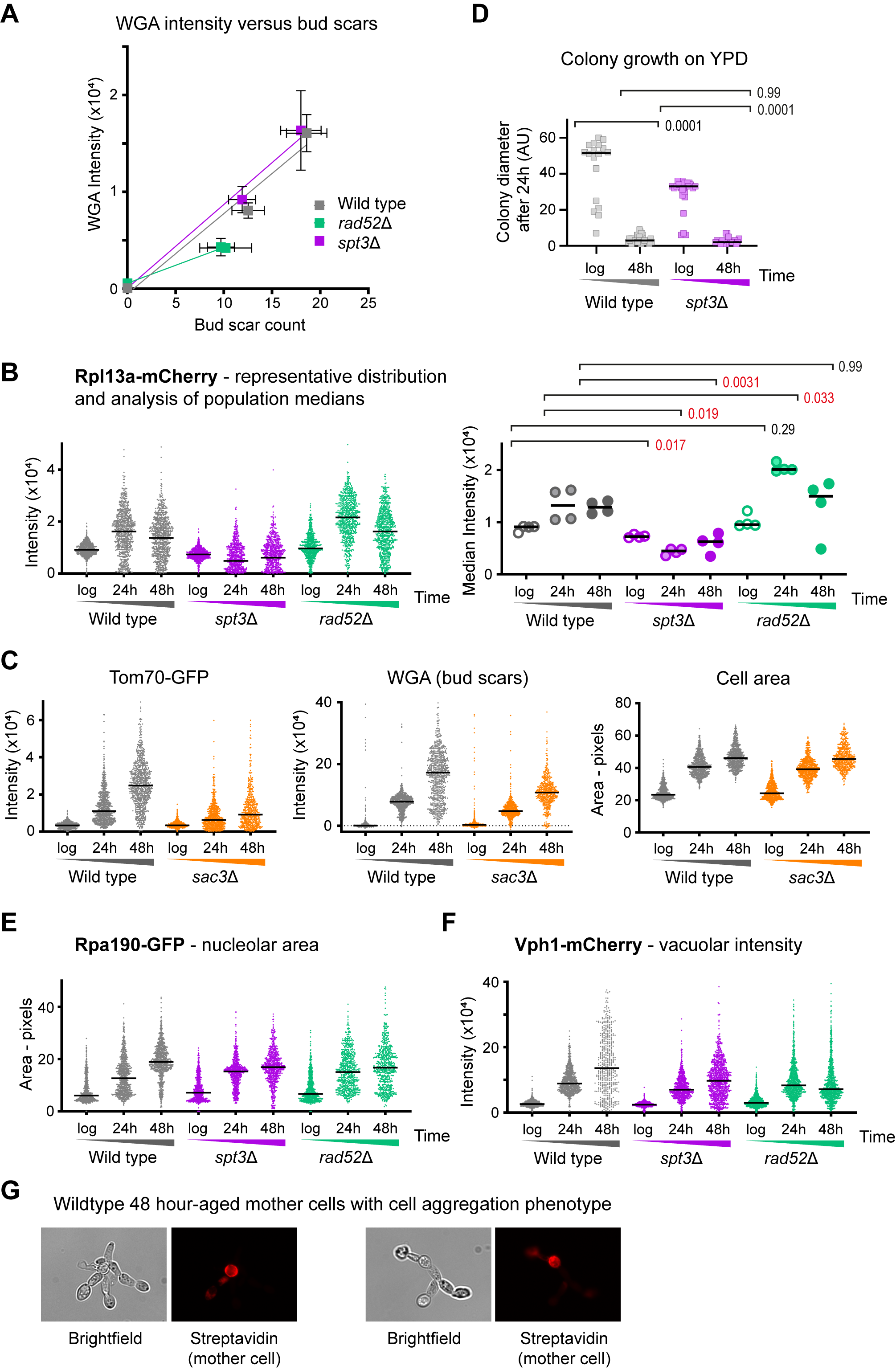

Supplement: S3 Fig — (A) Comparison between manual bud scar counts and median WGA intensities for wild type, spt3Δ, and rad52Δ at 24- and 48-hour time points. n = 15 cells for bud scars; n = 4 medians of biological replicate populations for WGA; error bars ±1 SD. (B) Analysis of Rpl13a-mCherry to show ribosomal protein abundance. Samples and analysis as in Fig 3A. (C) Plots of Tom70-GFP, Rpl13a-mCherry, WGA, and cell size for wild-type and sac3Δ cells at indicated ages, obtained as in Fig 3A. (D) Colony size assays measuring fitness of wild type and spt3Δ cells aged in glucose. Log phase cells and cells aged 48 hours then purified live in media were placed on YPD plates by micromanipulation. Colony diameters were measured on the micromanipulator screen after 24 hours. Only cells that formed visible colonies after a further 3 days were included in the analysis. Log phase cells were taken directly from culture and colony growth measured under the same conditions. p-Values calculated by Kruskal–Wallis test, n = 18 for log wild type, 25 for log spt3Δ, 40 for aged wild type, 22 for aged spt3Δ. (E) Distribution of signal areas from flow cytometry images of Rpa190-GFP in populations of log phase cells or mother cells aged for 24 and 48 hours. A total of 1,000 cells were imaged per population after gating for circularity (all) and biotin (aged cells only, based on streptavidin-Alexa647); images were post-filtered for focus of Rpa190-GFP. (F) Distribution of signal intensities from flow cytometry images of Vph1-mCherry in populations of log phase cells or mother cells aged for 24 and 48 hours. A total of 1,000 cells were imaged per population after gating for circularity (all) and biotin (aged cells only, based on streptavidin-Alexa647); images were post-filtered for focus of Vph1-GFP. (G) Example images of unseparated clusters of cells (brightfield) originating from single aged mother cells (streptavidin stained, red). The numerical data underlying this Figure can be found in S8 File. ERC, extr [file pbio.3002250.s003.tif]

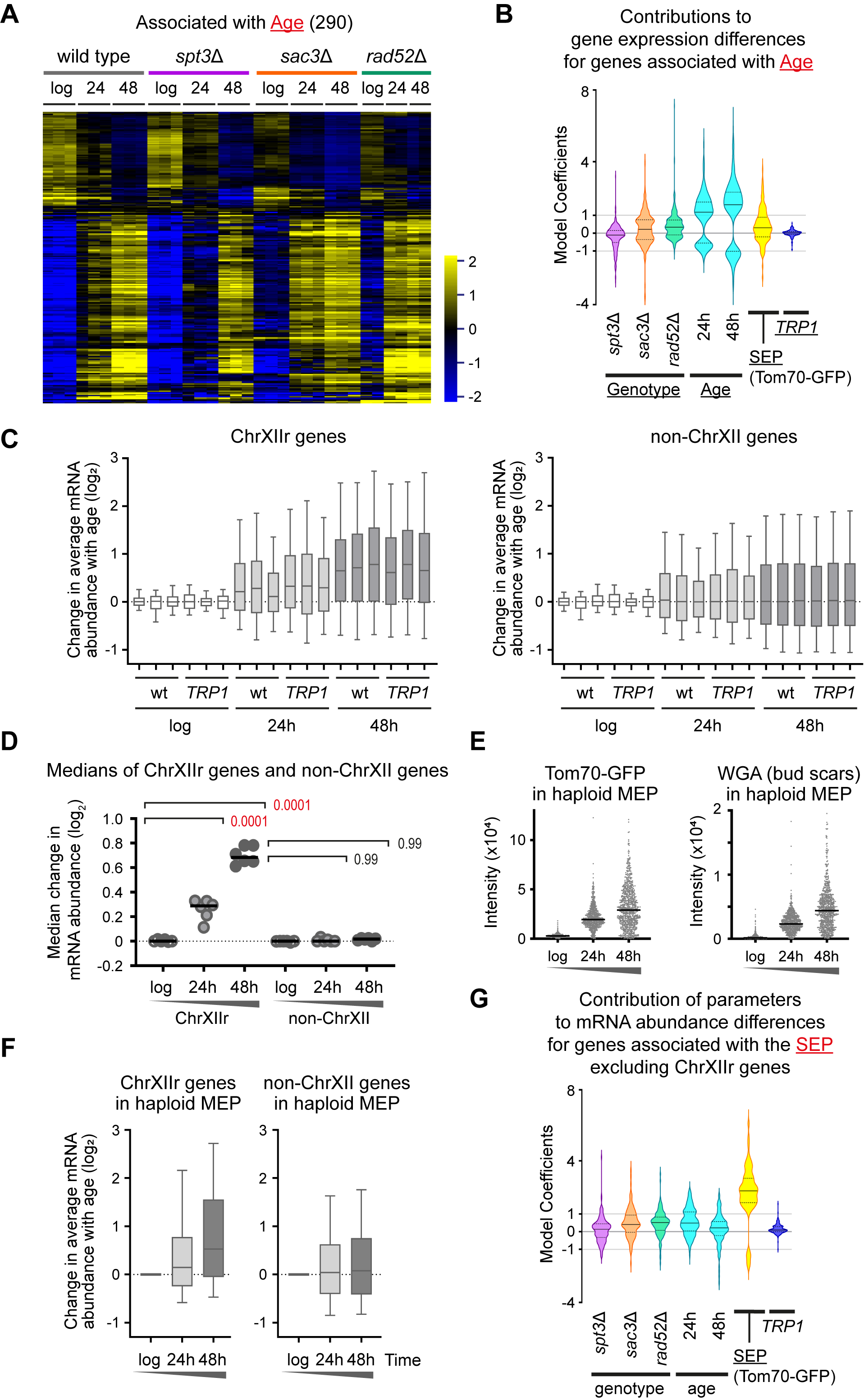

Supplement: S4 Fig — (A) Hierarchical clustering of log2 mRNA abundance for 290 genes called by DESeq2 Linear Model 2 as significantly different between datasets based on age. DESeq2 modelling as described for 4B, clustering procedure as for 2B. Individual biological replicates shown (3 for wild type, spt3Δ, and sac3Δ and 2 for rad52Δ). (B) Coefficients representing the contribution of each individual parameter to the differences in mRNA abundance of the set of genes significantly differentially expressed with Tom70-GFP levels across all datasets calculated using DEseq2 linear model 2 as described for 4B. (C) Log2 change of mRNA abundance from log phase to given time points for all genes on ChrXIIr (left) or all genes on chromosomes other than XII (right). Individual biological replicates are shown; boxes show median and interquartile range; whiskers show upper and lower deciles. (D) Medians of mRNA abundance change datasets (Fig 4C) for ChrXIIr and other chromosomes. Wild type and TRP1 datasets are pooled to give n = 6 per condition, p-values calculated by 1-way ANOVA with post hoc Tukey test. Red p-values indicate significance at p < 0.05. (E) Distribution of signal intensities from flow cytometry images of Tom70-GFP and WGA-Alexa405 in populations of haploid MATα log phase cells or mother cells aged for 24 and 48 hours in YPD. A total of 1,000 cells are imaged per population after gating for circularity (all) and biotin (aged cells only, based on streptavidin-Alexa647). Images are post-filtered for focus of Tom70-GFP, leaving approximately 600 cells per population. (F) Log2 change of mRNA abundance for haploid MATα MEP cells from log phase to given time points for all genes on ChrXIIr (left) or all genes on chromosomes other than XII (right). (G) As Fig 4B excluding genes on ChrXIIr. The numerical data underlying this Figure can be found in S8 File. MEP, mother enrichment program; SEP, senescence entry point. (TIF) [file pbio.3002250.s004.tif]

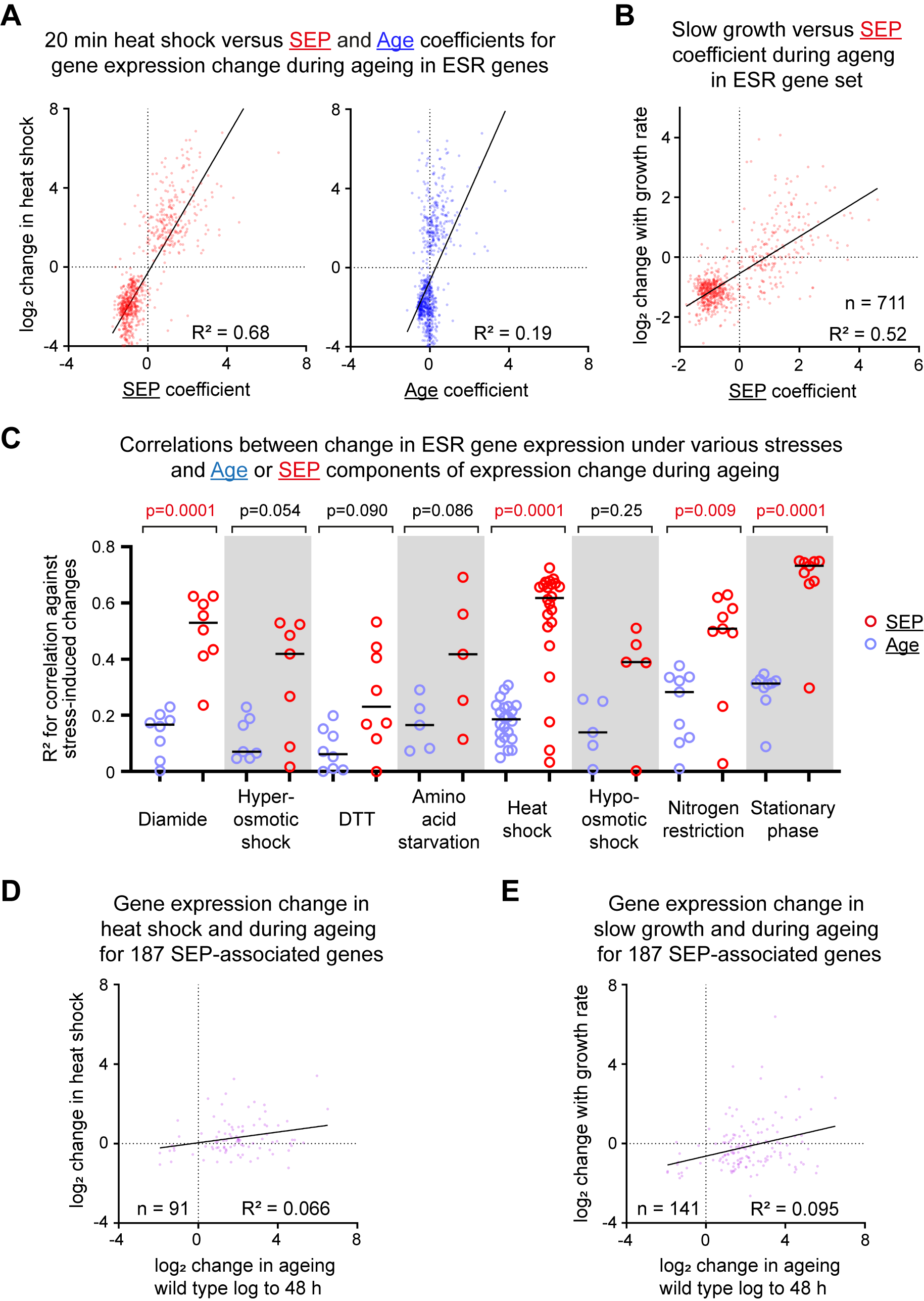

Supplement: S5 Fig — (A) Correlations between coefficients for SEP and Age, representing the contribution of these variables to gene expression change during ageing calculated by Linear Model 3, and gene expression change during a 20-minute heat shock (GSE18). Only genes participating in the ESR are included [13]. R2 values calculated by linear regression. (B) Correlation between coefficients for the SEP, representing the contribution of this variable to gene expression change during ageing calculated by Linear Model 3, and gene expression difference between specific growth rates μ = 0.1 and μ = 0.33 [66]. Only genes participating in the ESR are included [13]. R2 value calculated by linear regression; n is number of genes analysed as not all ESR genes were measured by Regenberg and colleagues. (C) Summary of correlations between coefficients for SEP and Age, representing the contribution of these variables to gene expression change during ageing calculated by Linear Model 3, and gene expression change during different stresses (GSE18). R2 values were calculated for the comparison of each individual stress dataset reported by Gasch and colleagues and the SEP/Age coefficients. R2 values were then binned into 8 different stress types, and p-values representing the differences between correlations to the SEP and Age coefficients calculated by 1-way ANOVA. (D) Correlation between gene expression change in ageing (wild type log phase to 48 hours) and in heat shock (GSE18) for the set of 187 genes significantly associated with the SEP by Linear Model 2. R2 value calculated by linear regression; n is number of genes analysed as not all the 187 SEP genes were measured by Gasch and colleagues. (E) Correlation between gene expression change in ageing (wild-type log phase to 48 hours) and between specific growth rates μ = 0.1 and μ = 0.33 [66] for the set of 187 genes significantly associated with the SEP by Linear Model 2. R2 value calculated by linear regression; n is number of genes analysed as [file pbio.3002250.s005.tif]

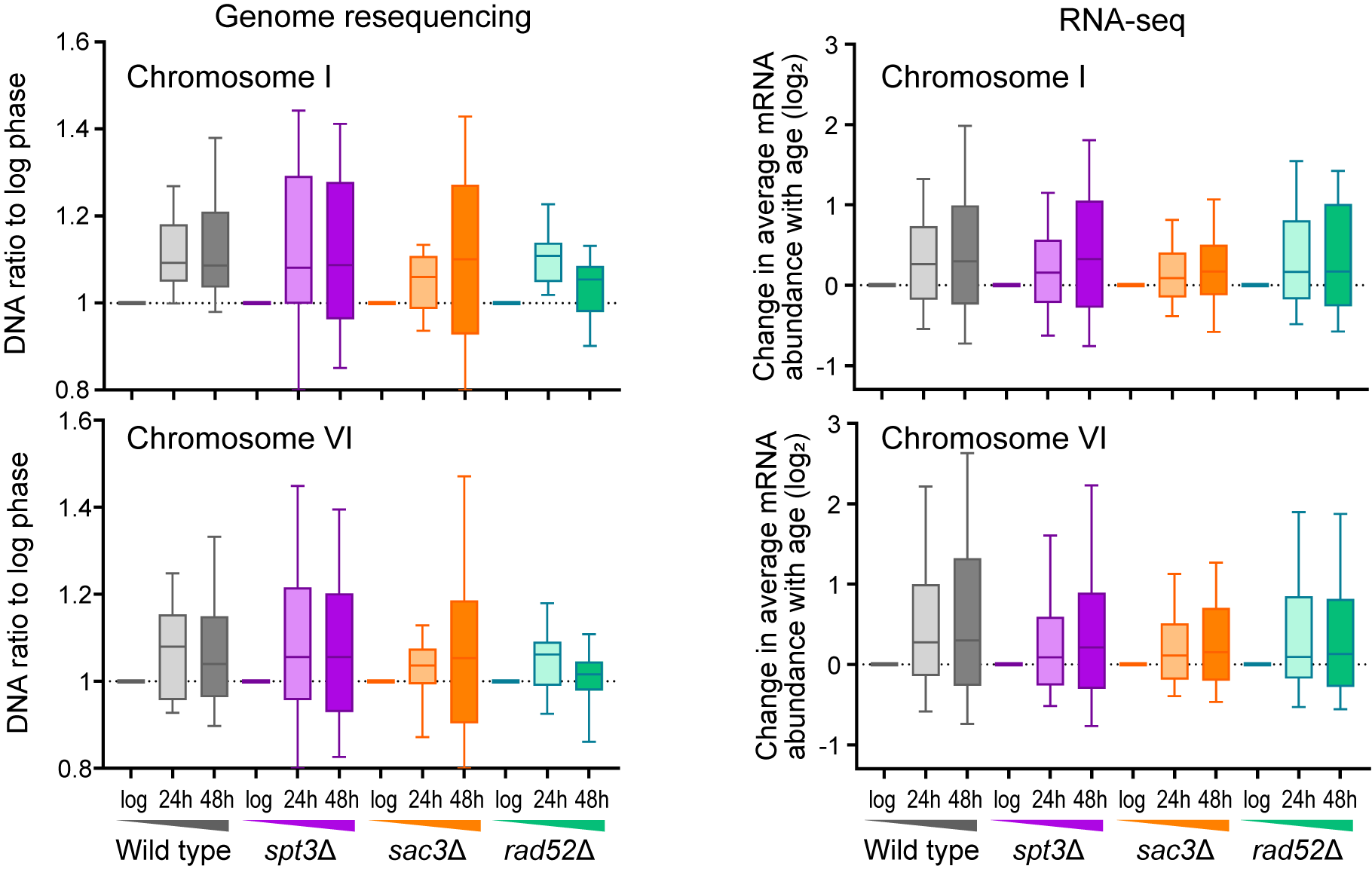

Supplement: S6 Fig — Genome resequencing data and RNA-seq data as in Figs 5C and 4C, respectively, showing mild accumulations of Chromosomes I and VI during ageing. The numerical data underlying this Figure can be found in S8 File. (TIF) [file pbio.3002250.s006.tif]

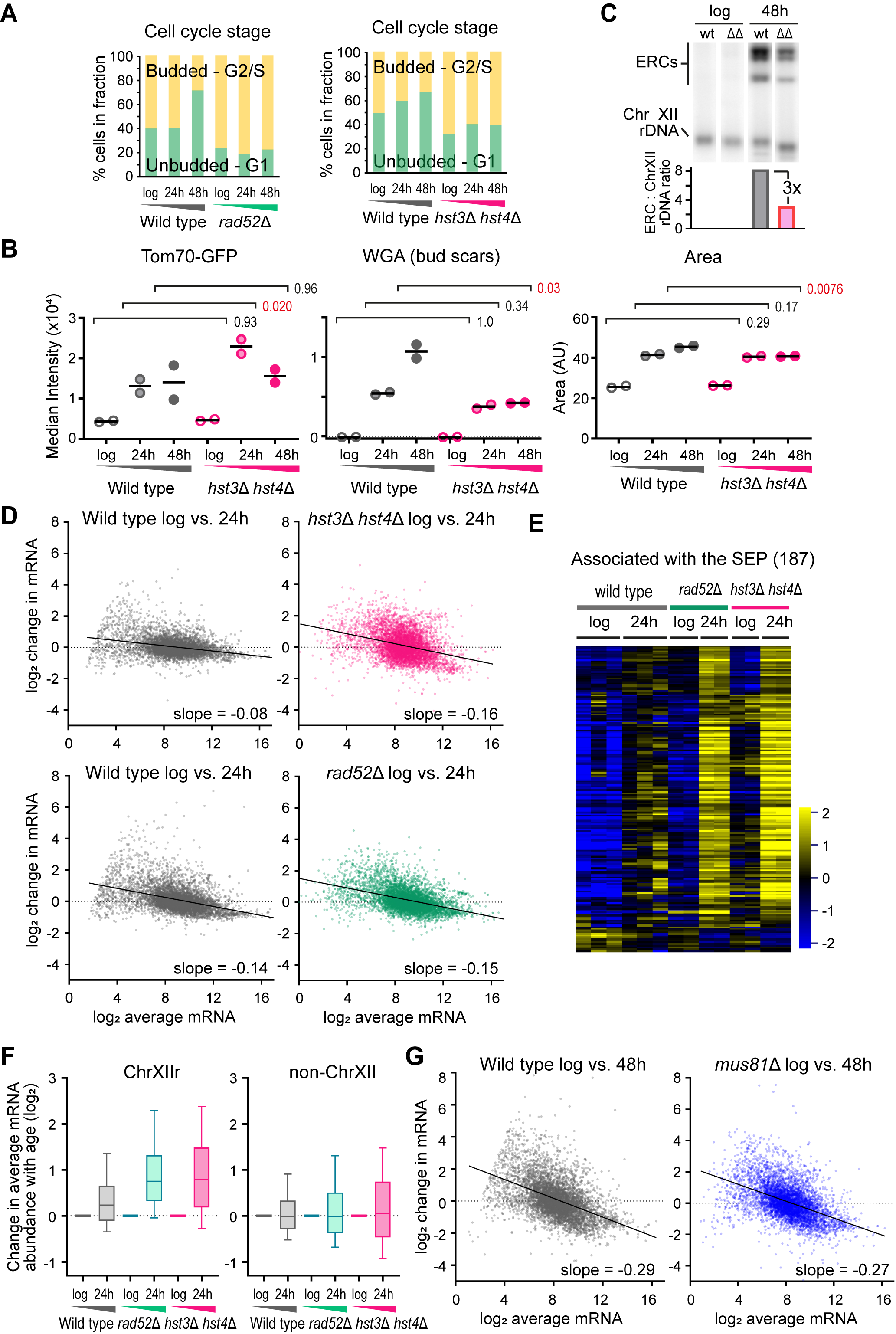

Supplement: S7 Fig — (A) Cell cycle stage plots comparing the percentage of budded and unbudded cells. Imaging flow cytometry was performed with gating for streptavidin (aged samples only), then budded and unbudded cells called based on circularity and validated by examining representative images. (B) Imaging flow cytometry analysis of Tom70-GFP intensity, which increases hugely in G1 cells after the SEP [5], WGA, which reports on replicative age, and cell size in wild type and hst3Δ hst4Δ. Cells were gated for streptavidin staining (aged samples only), which filters out young cells, then for circularity, which selects towards G1 cells and removes clumps. Graph shows medians of biological replicate populations; p-values calculated from 2-way ANOVA with post hoc Tukey test using genotype and age as independent variables, n = 2. Red p-values indicate significance at p < 0.05. (C) Southern blot analysis of ERC abundance in log phase and 24-hour-aged wild-type and hst3Δ hst4Δ MEP cells; quantification shows ration of ERC bands to chromosomal rDNA. Individual lanes were spliced from the same blot image; no differential image processing was applied. (D) MA plots comparing log2 mRNA abundance distributions between log phase and 48-hour-aged samples from wild type and indicated mutants. x-Axis is log2 average normalised read count; y-axis is change in log2 normalised read count from young to old. Slope is calculated by linear regression. Different matched wild-type control data are presented for each mutant; rad52Δ data are provided as a comparison for hst3Δ hst4Δ. Data for each genotype and age calculated as the log2 mean normalised read counts per gene of 2 biological replicates for mutants, 3 biological replicates for wild type. (E) Hierarchical clustering of log2 mRNA abundance for 187 genes called by a DESeq2 Linear Model 2 as significantly different between datasets based on the SEP, as in Fig 4A, for wild type, rad52Δ, and hst3Δ hst4Δ at log and 24 hours, 2 biological replicates per time [file pbio.3002250.s007.tif]

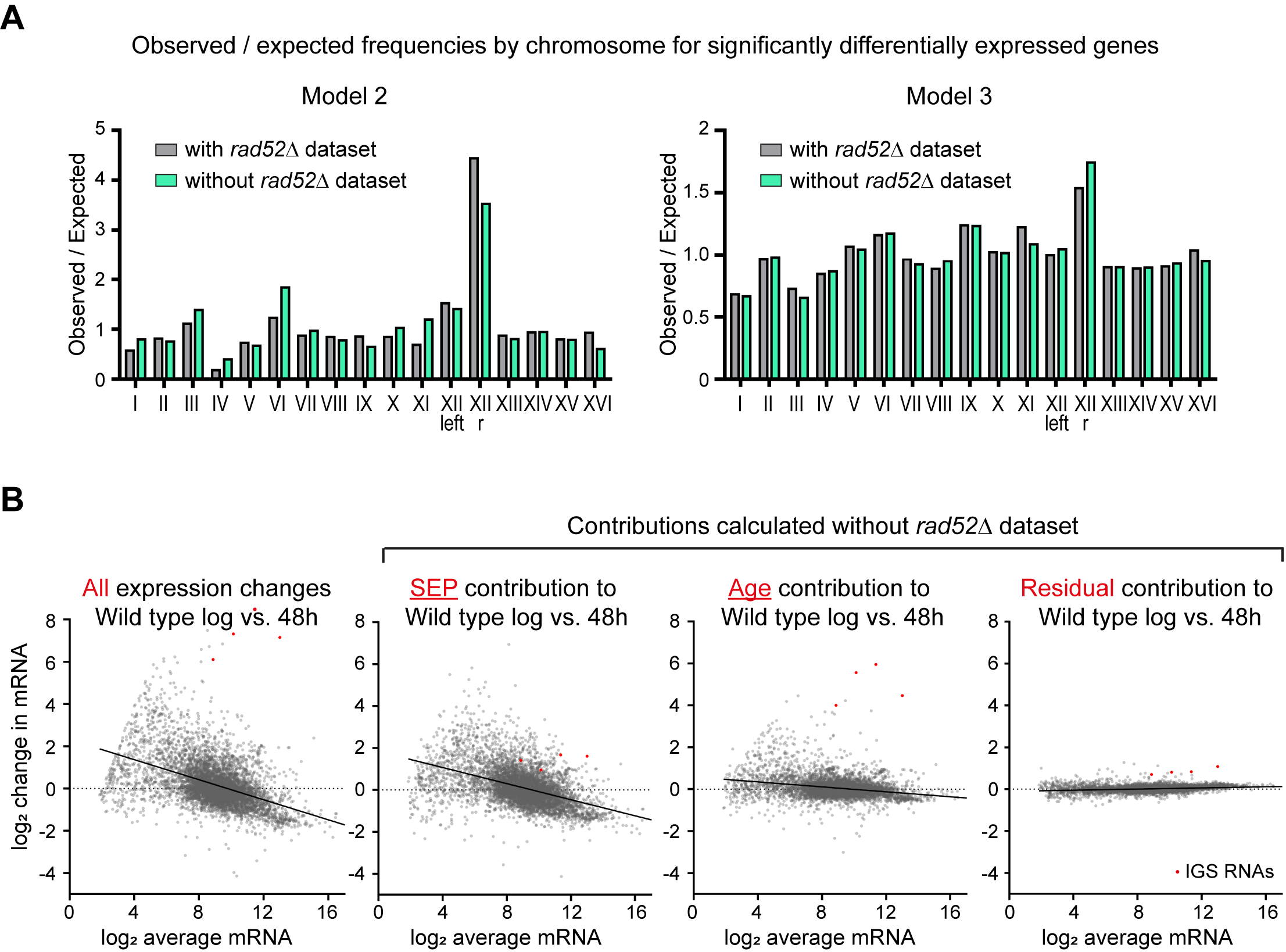

Supplement: S8 Fig — (A) Fraction of genes significantly differentially expressed based on SEP variable discovered by Linear Models 2 and 3 fitted using either the full dataset of wild type, spt3Δ, sac3Δ, and rad52Δ, or a reduced dataset of just wild type, spt3Δ, and sac3Δ. Values are observed number of genes per chromosome divided by the expected number of genes (calculated from the total number of significantly differentially expressed genes × genes on chromosome / genes in genome). Chromosome XII values are split between XII left–the region from the left telomere to the rDNA, and XIIr–from the rDNA to the right telomere (ChrXIIr). Full output of the models is provided in S4 File and S5 File. (B) Assignment of gene expression change in wild-type ageing log to 48 hours to SEP and Age contributions calculated by Linear Model 3 fitted without rad52Δ dataset, displayed as MA plots. Analysis as in Fig 4F; left panel shows wild-type ageing data for comparison and is identical to the left-hand plot in Fig 4F. The numerical data underlying this Figure can be found in S8 File. (TIF) [file pbio.3002250.s008.tif]

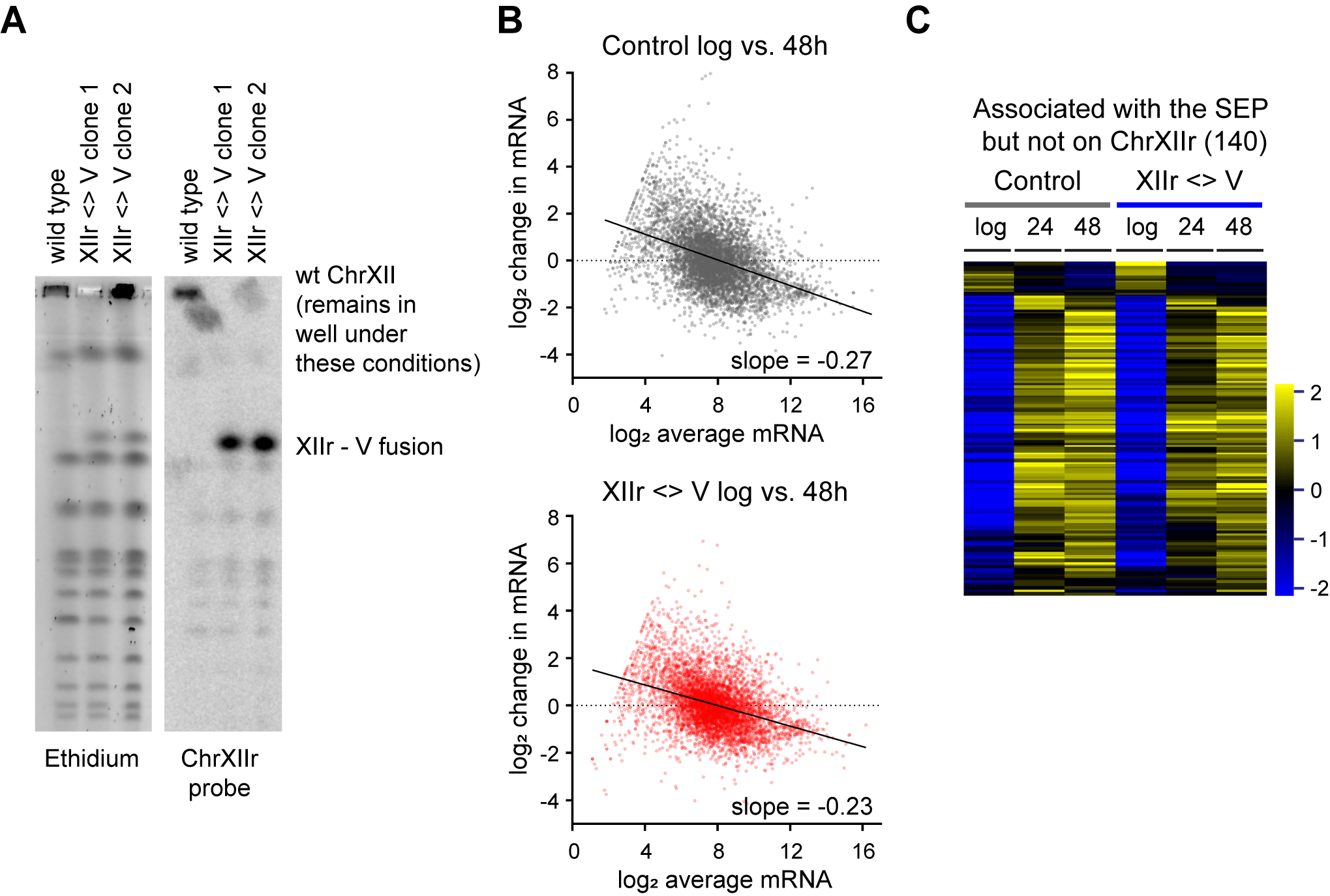

Supplement: S9 Fig — (A) Pulsed field gel electrophoresis analysis showing successful translocation of ChrXIIr onto chromosome V. (B) MA plots comparing log2 mRNA abundance distributions between log phase and 48-hour-aged samples from a:a diploid wild-type and Chr XII <>V translocation strain. x-Axis is log2 average normalised read count; y-axis is change in log2 normalised read count from young to old. Slope is calculated by linear regression. (C) Hierarchical clustering of log2 mRNA abundance for 187 genes called by a DESeq2 Linear Model 2 as significantly different between datasets based on the SEP, as in Fig 4A, excluding genes on ChrXIIr, for a: a diploid wild-type and Chr XII <>V translocation strain. The numerical data underlying this Figure can be found in S8 File. rDNA, ribosomal DNA; SEP, senescence entry point. (TIF) [file pbio.3002250.s009.tif]

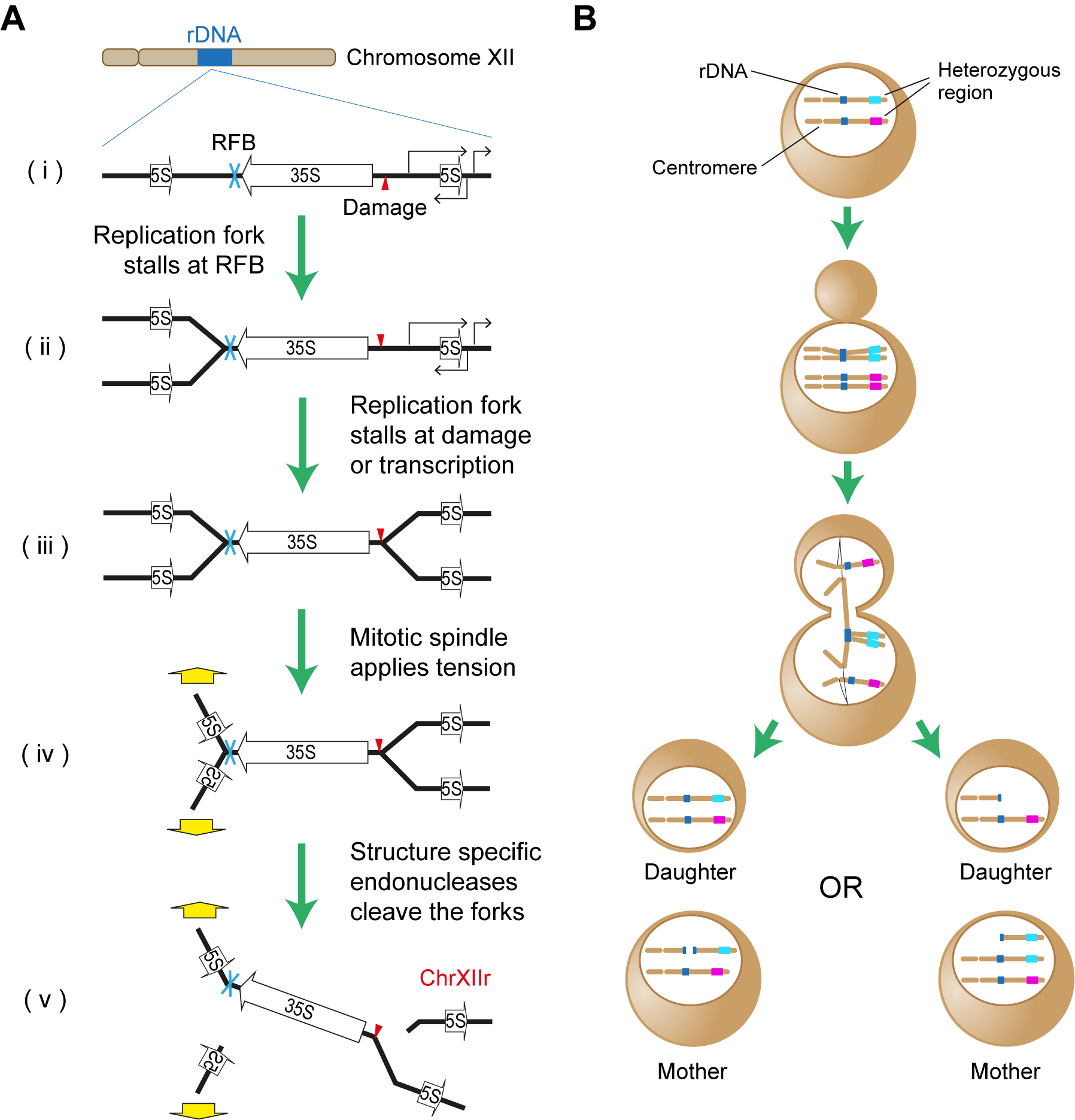

Supplement: S10 Fig — (A) Replication fork dynamics leading to incomplete replication at anaphase. (i) Potential replication fork stalling elements in the rDNA include the RFB, DNA damage, and regions highly expressed by RNA polymerase I, II, and III. (ii) First replication fork stalls at the RFB or on collision with RNA polymerase I. (iii) Converging replication fork stalls due to DNA damage, collision with transcription units, topological strain, etc. (iv) Centromeres are pulled apart on the mitotic spindle, but segregation cannot be completed. (v) Cleavage of replication fork structures by structure-specific endonucleases allows segregation to complete but forms ChrXIIr. (B) ChrXIIr formation in the context of heterozygous markers. Incomplete replication leads to one chromosome forming a bridging chromosome, resolution of which either leaves both chromosome fragments in one nucleus (left), in which case the break can be repaired, or separates the fragments (right) in which case ChrXIIr formation in the mother is accompanied by loss of heterozygosity in the daughter. Further recombination events between ChrXIIr and full-length chromosome XII in the mother can lead to loss of heterozygosity also in the mother. rDNA, ribosomal DNA; RFB, replication fork barrier. (TIF) [file pbio.3002250.s010.tif]

Change in abundance with age of all chromosomes other than XII

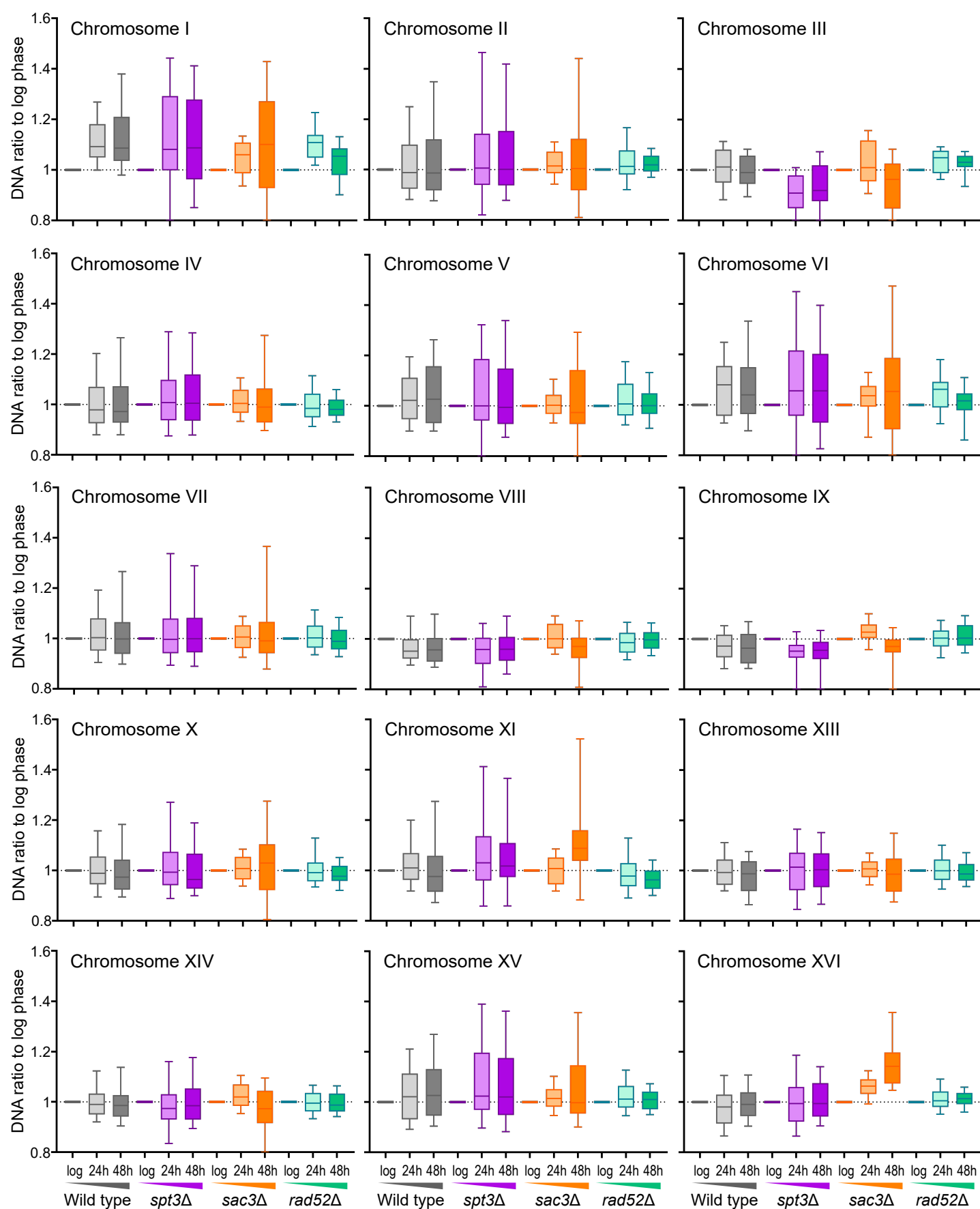

Supplement: S4 File — Change in age for individual chromosomes in each mutant, data processed as in Fig 5C. Scales have been kept the same as in Fig 5C to allow easy comparison. (PDF) [file pbio.3002250.s017.pdf]
