# Supplementary material for: Senescence in yeast is associated with amplified linear fragments of chromosome XII rather than ribosomal DNA circle accumulation
Source: PLoS Biol. 2023 Aug 29;21(8):e3002250. doi: 10.1371/journal.pbio.3002250 (PMC10464983; doi:10.1371/journal.pbio.3002250)
Supplement: S3 Table — (DOCX) [file pbio.3002250.s013.docx]

| pJH9 | pFA6a TRP1 | [1] |  |
| --- | --- | --- | --- |
| pJH20 | pFA6a GFP HIS3MX6 | [1] |  |
| pJH369 | pAW8-mCherry | [2] |  |
| pJH388 | pFA6a-GFP-KanMX6 no I-SceI | I-SceI site removed by site directed mutagenesis | |
| pJH381 | pGSTU | delitto perfetto plasmid with Kan swapped out for TRP1 | |
| pJH387 | pGST-V-U | For reciprocal translocations with Chr V sub-telomere. Has region of V:564025-566025 inserted between URA3 and TRP1 markers with SceI site in the middle. Note plasmid yield was weirdly low | |
| pJH73 | pFA6a URA3 | [3] |  |

**Refernces**

1. Longtine MS, McKenzie A, 3rd, Demarini DJ, Shah NG, Wach A, Brachat A, et al. Additional modules for versatile and economical PCR-based gene deletion and modification in Saccharomyces cerevisiae. Yeast. 1998;14(10):953-61. PubMed PMID: 9717241.

2. Watson AT, Garcia V, Bone N, Carr AM, Armstrong J. Gene tagging and gene replacement using recombinase-mediated cassette exchange in Schizosaccharomyces pombe. Gene. 2008;407(1-2):63-74. Epub 2007/12/07. doi: 10.1016/j.gene.2007.09.024. PubMed PMID: 18054176.

3. Houseley J, Tollervey D. Repeat expansion in the budding yeast ribosomal DNA can occur independently of the canonical homologous recombination machinery. Nucleic Acids Res. 2011;39(20):8778-91. Epub 2011/07/20. doi: 10.1093/nar/gkr589. PubMed PMID: 21768125; PubMed Central PMCID: PMC3203602.
