# Supplementary material for: Senescence in yeast is associated with amplified linear fragments of chromosome XII rather than ribosomal DNA circle accumulation
Source: PLoS Biol. 2023 Aug 29;21(8):e3002250. doi: 10.1371/journal.pbio.3002250 (PMC10464983; doi:10.1371/journal.pbio.3002250)
Supplement: S1 Raw Images — (PDF) [file pbio.3002250.s022.pdf]

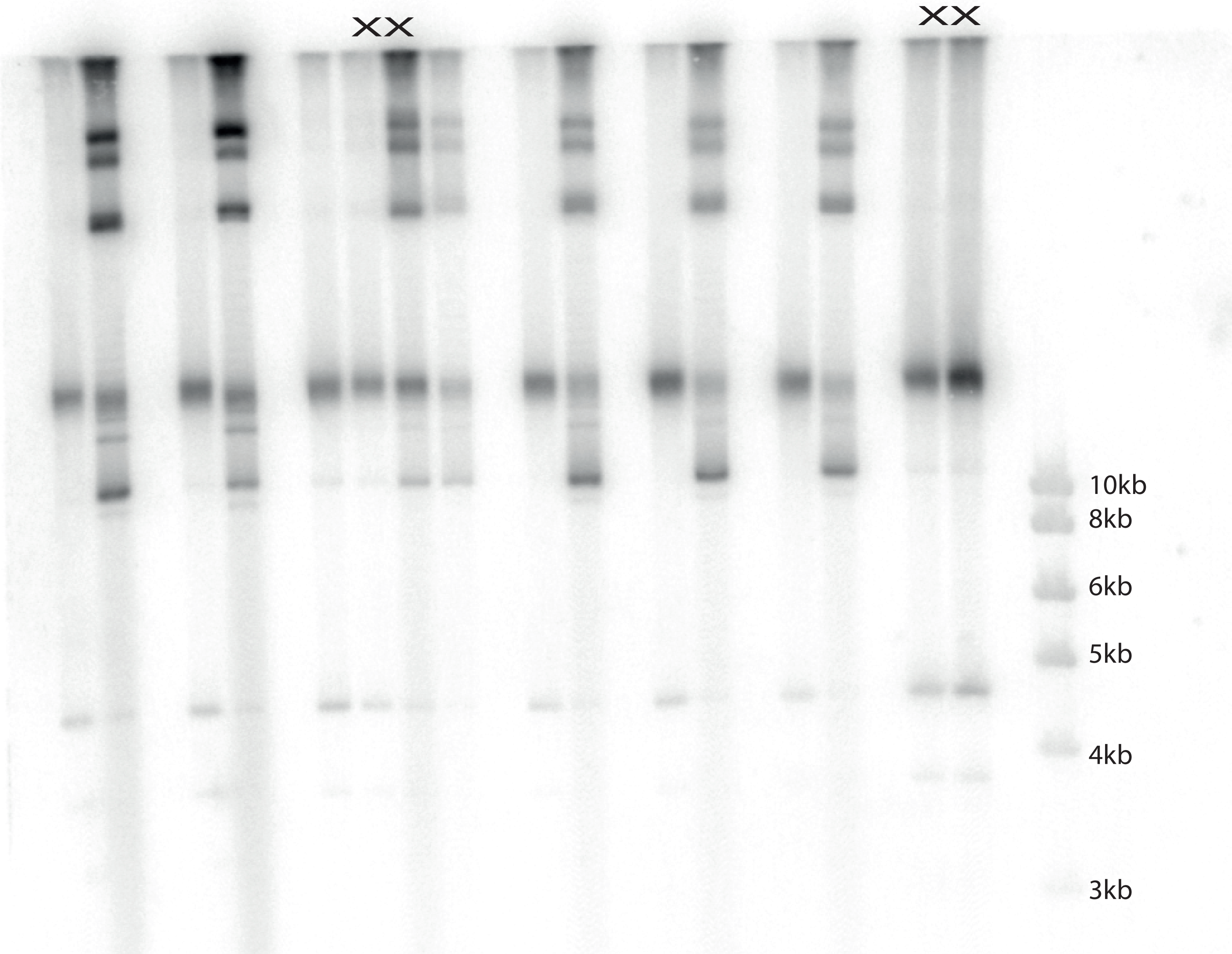

Genomic DNA digested with XhoI, separated on1% agarose gel, blotted and probed with a 32P random primed probe against the intergenic spacer region of the rDNA. Probe was detected with a Typhoon FLA3000 phosphorimager

Sample order:

wt glucose log
wt glucose 48h

wt glucose log
wt glucose 48h

wt glucose log
wt glucose 6h
wt glucose 24h
wt glucose 48h

fob1 glucose log
fob1 glucose 48h

fob1 glucose log
fob1 glucose 48h

fob1 glucose log
fob1 glucose 48h

wt log
wt log

Used to generate Figure S1A

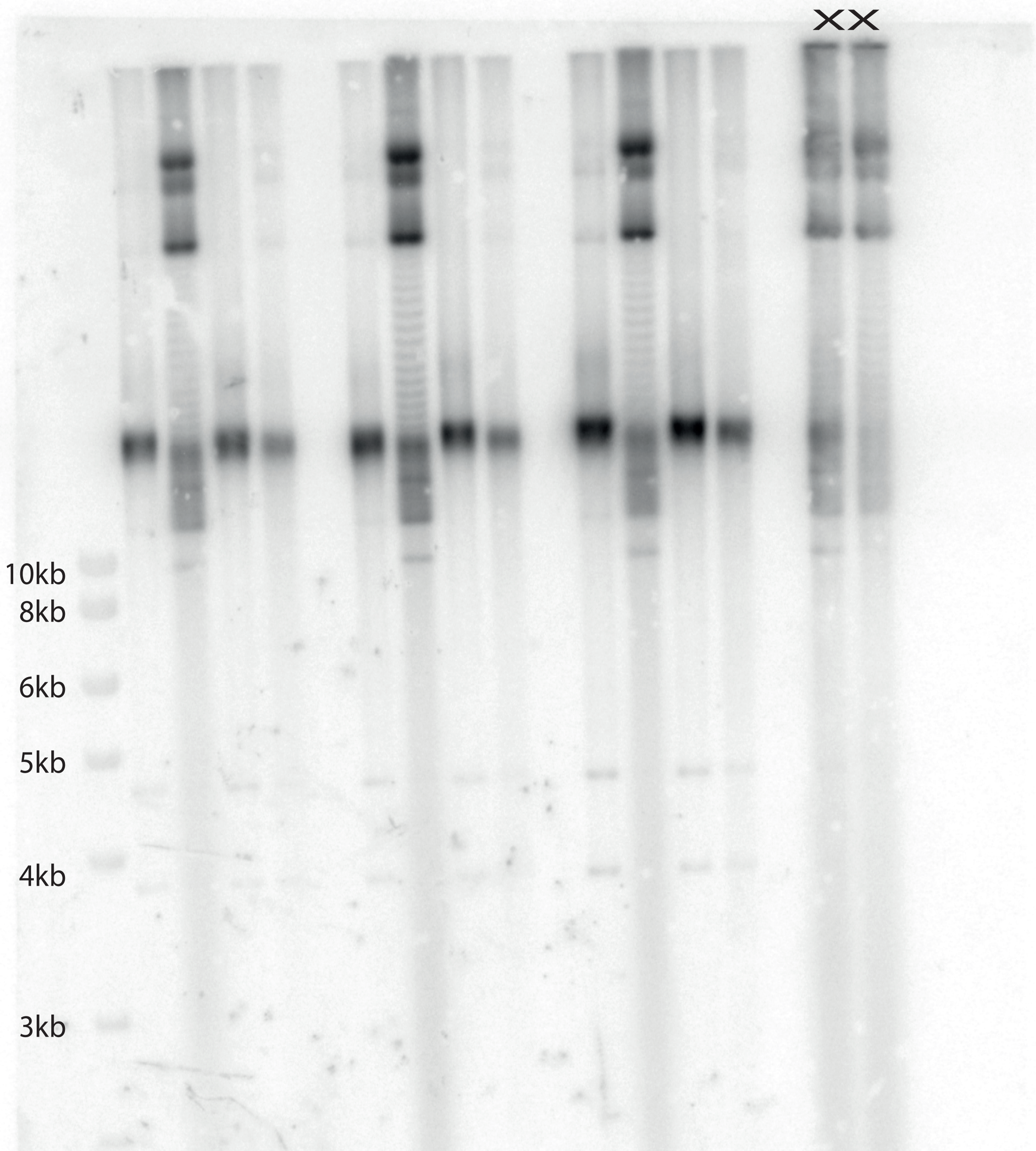

Genomic DNA digested with XhoI, separated on1% agarose gel, blotted and probed with a 32P random primed probe against the intergenic spacer region of the rDNA. Probe was detected with a Typhoon FLA3000 phosphorimager

Sample order:

wt glucose log
wt glucose 48h
rad52 glucose log
rad52 glucose 48h

wt glucose log
wt glucose 48h
rad52 glucose log
rad52 glucose 48h

wt glucose log
wt glucose 48h
rad52 glucose log
rad52 glucose 48h

unused
unused

Used to generate Figure S1B left

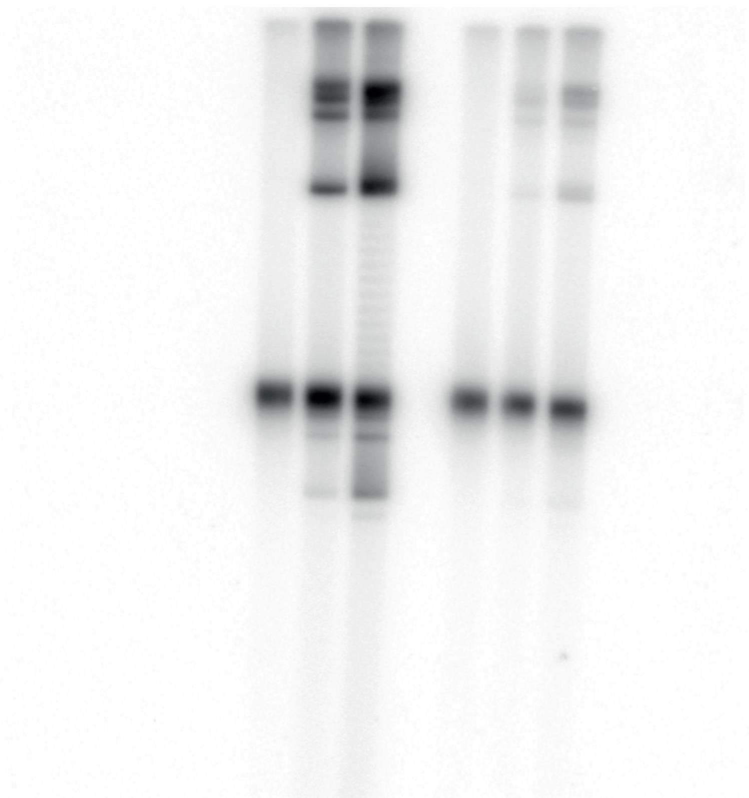

Genomic DNA digested with XhoI, separated on1% agarose gel, blotted and probed with a 32P random primed probe against the intergenic spacer region of the rDNA. Probe was detected with a Typhoon FLA3000 phosphorimager

Sample order:

wt glucose log
wt glucose 24h
wt glucose 48h

spt3 glucose log
spt3 glucose 24h
spt3 glucose 48h

Used to generate Figure S1B middle

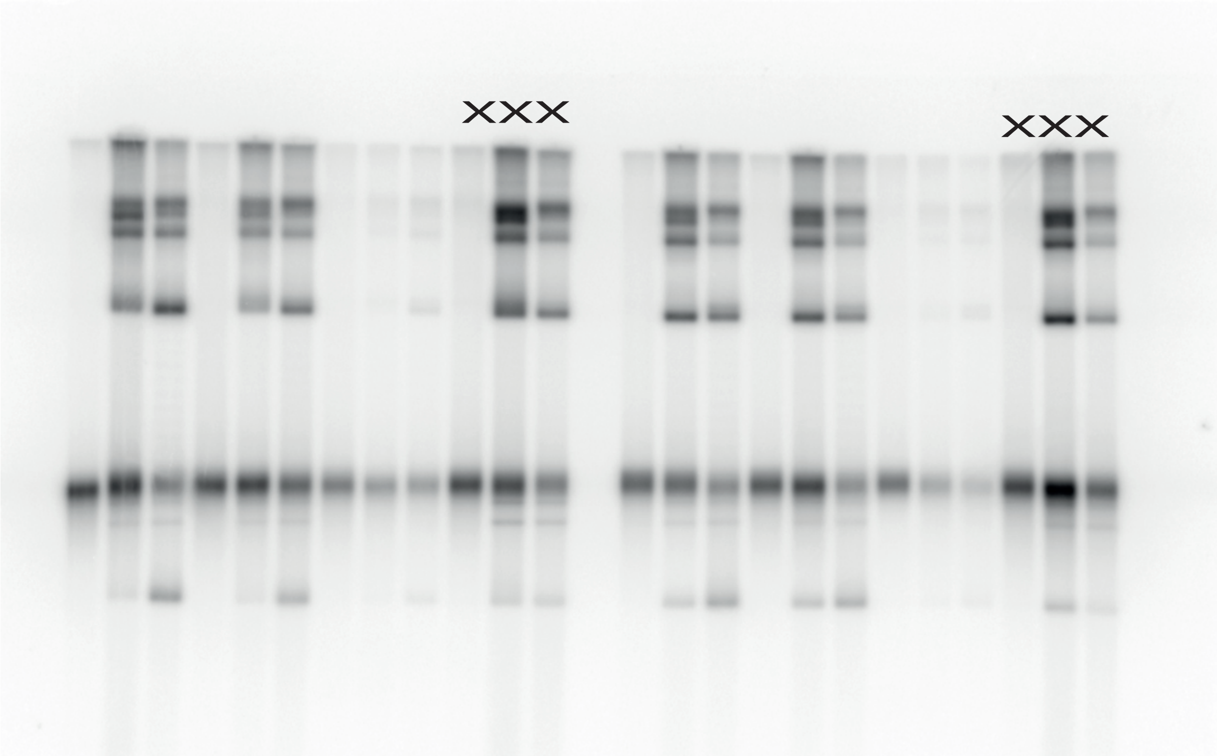

Genomic DNA digested with XhoI, separated on1% agarose gel, blotted and probed with a 32P random primed probe against the intergenic spacer region of the rDNA. Probe was detected with a Typhoon FLA3000 phosphorimager

Sample order:

wt glucose log
wt glucose 24h
wt glucose 48h

TRP1 glucose log
TRP1 glucose 24h
TRP1 glucose 48h

sac3 glucose log
sac3 glucose 24h
sac3 glucose 48h

rif1 glucose log
rif1 glucose 24h
rif1 glucose 48h

Used to generate Figure S1B right

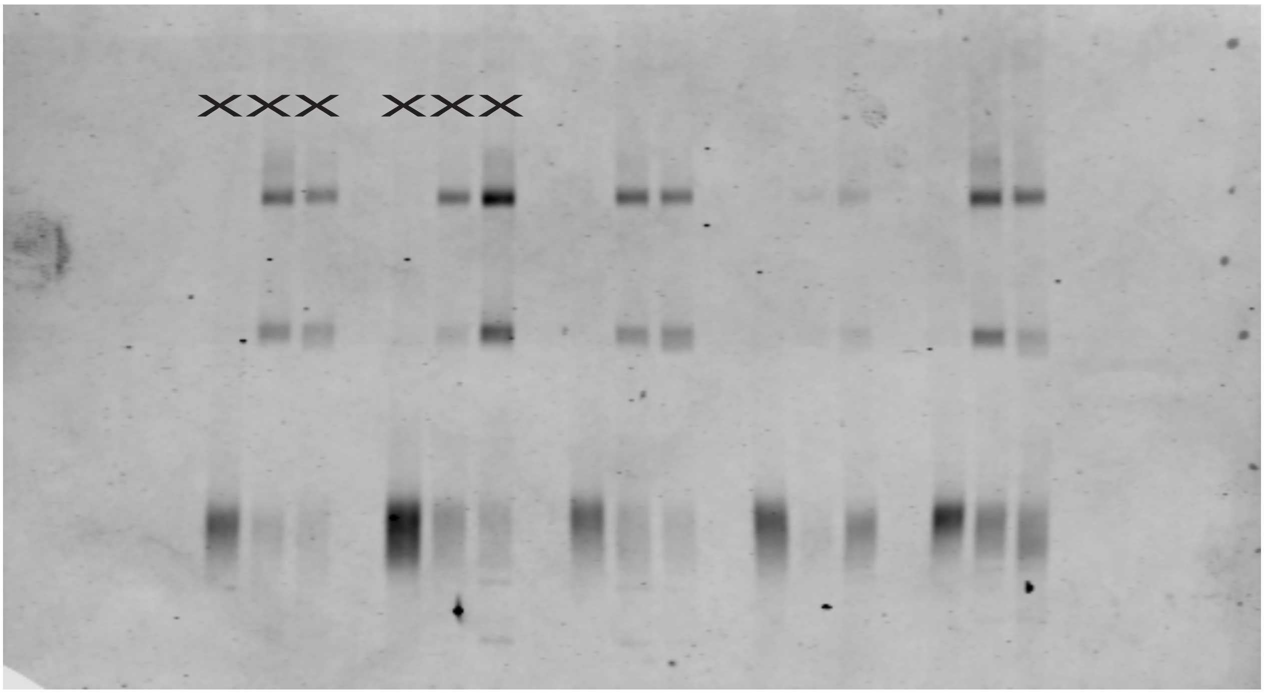

Genomic DNA digested with XhoI, separated on1% agarose gel, blotted and probed with a biotinylated RNA probe against the intergenic spacer region of the rDNA. Probe was detected with IRDye680 Streptavidin and scanned on a Licor Odyssey CLx

Sample order:

wt glucose log
wt glucose 24h
wt glucose 48h

wt galactose log
wt galactose 24h
wt galactose 48h

wt glucose log
wt glucose 24h
wt glucose 48h

mus81 glucose log
mus81 glucose 24h
mus81 glucose 48h

yen1 glucose log
yen1 glucose 24h
yen1 glucose 48h

Used to generate Figure 6A

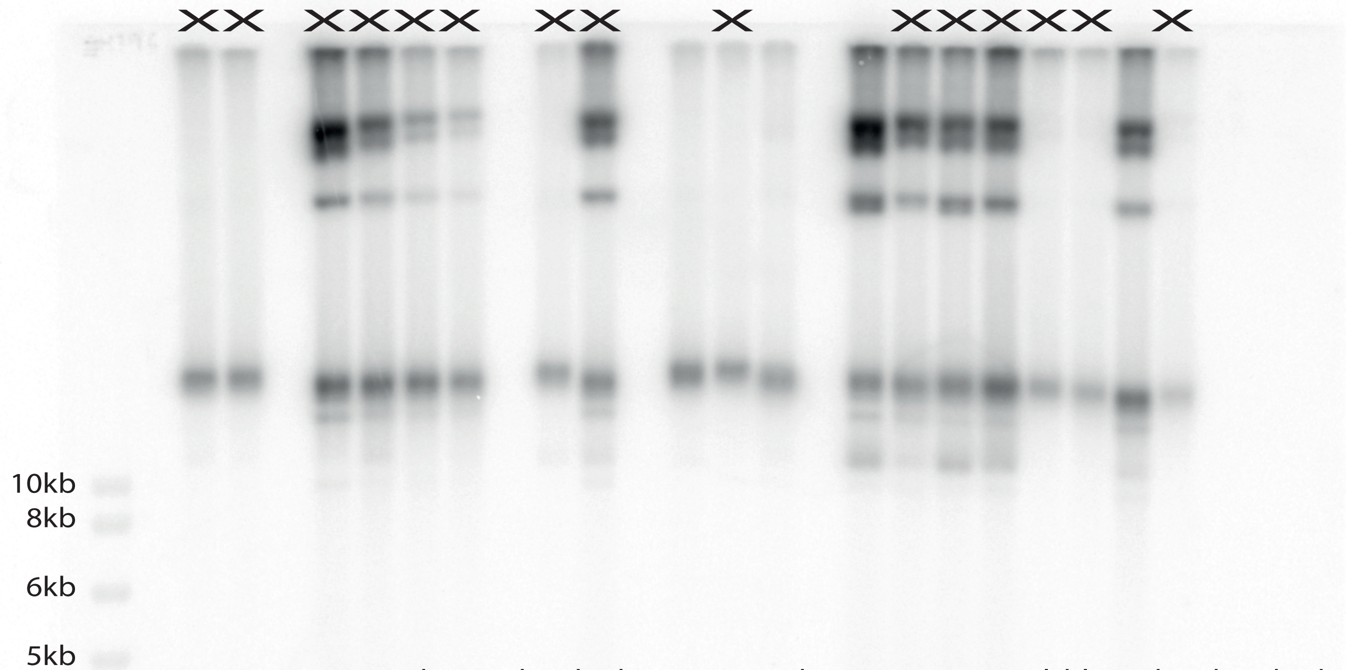

Genomic DNA digested with XhoI, separated on1% agarose gel, blotted and probed with a 32P random primed probe against the intergenic spacer region of the rDNA. Probe was detected with a Typhoon FLA3000 phosphorimager

Sample order:

wt glucose log
mus81 glucose log

wt glucose 24h
wt glucose 24h+1mM Cu
mus81 glucose 24h
mus81 glucose 24h+1mM Cu

pol32 glucose log
pol32 glucose 24h

wt glucose log
asf1 glucose log
hst3 hst4 glucose log

wt glucose 24h
wt glucose 24h+1mM Cu
wt glucose 24h+nicotinamide
wt glucose 24h+1mM Cu+nicotinamide

asf1 glucose 24h
asf1 glucose 24h+1mM Cu

hst3 hst4 glucose 24h
hst3 hst4 glucose 24h+1mM Cu

Used to generate Figure S7B

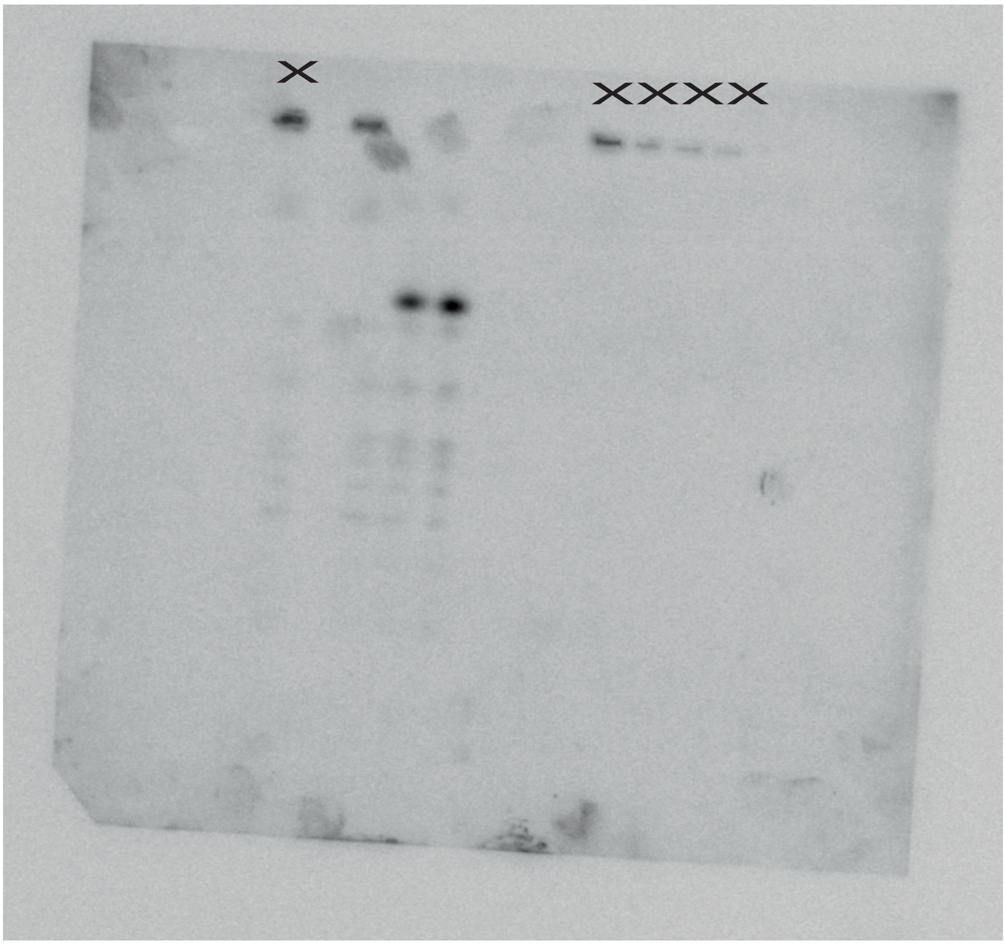

Genomic DNA separated onPFGE gel (see Methods), blotted and probed with a 32P random primed probe against BNA5. Probe was detected with a Typhoon FLA3000 phosphorimager.

Sample order:

wt

wt
ChrXII <> V clone 1
ChrXII <> V clone 2

Used to generate Figure S9A
