# Supplementary material for: Senescence in yeast is associated with amplified linear fragments of chromosome XII rather than ribosomal DNA circle accumulation
Source: PLoS Biol. 2023 Aug 29;21(8):e3002250. doi: 10.1371/journal.pbio.3002250 (PMC10464983; doi:10.1371/journal.pbio.3002250)
Supplement: Attachment — Submitted filename: zylstra et al response to reviewers - AZ.docx [file pbio.3002250.s023.docx]

**Response to reviewer’s comments (Zylstra *et al*):**

**Reviewer #1:**

Numerous studies in budding yeast have demonstrated a strong correlation between aging and the accumulation of extra-chromosomal ribosomal circles (ERCs). Indeed, high levels of ERCs are widely believed to be the proximal driver of senescence, although a causal link remains unsubstantiated. In the current manuscript, the authors use mutants that are incapable of either generating or accumulating ERCs to explore the issue of causality. They show convincingly that ERC accumulation is separable from transcriptional and microscopic hallmarks of replicative aging. Furthermore, their transcriptional analyses, combined with previously published work, led them to identify a fragment of the right arm of chrXII (chrXIIr) that can arise due to replication difficulties at the rDNA. The authors show that increased levels of chrXIIr are more tightly associated with hallmarks of aging than are high levels of ERCs. This is not only interesting in itself, but it also makes discoveries about the biology of aging in yeast more relevant to that in higher eukaryotes, which don't accumulate ERCs.

The work is done carefully, and the topic will be of broad interest to readers of PLoS Biology. My major concern with the manuscript is that it left me unclear about what the authors believe to be the causal relationship between the chrXIIr fragment and replicative senescence. They have clearly demonstrated that hallmarks of aging can occur in the absence of ERC accumulation, thereby proving that ERC accumulation is not required for aging. They have also shown a correlation between appearance of the chrXIIr fragment and aging phenotypes. However, as far as I can tell, just as they argue that correlation does not imply causation with ERCs, the same thing can be argued with the chrXIIr fragment. (The chrXIIr fragment is more tightly associated with the senescence entry program, or SEP, than are ERCs, but the SEP has not been demonstrated to be causal for age-related death.) This in no way nullifies the importance and novelty of the current work, but the authors must explain their model better.

Having re-read the Discussion we agree with the reviewer that we had focused overly on the formation of ChrXIIr and resolution of the ChrXIIr model with existing literature without actually stating our model for how ChrXIIr is involved in the ageing process. We have re-organised the Discussion such that first subsection provides a clear statement of the new model, including text that was previously rather buried right at the end, and added a new schematic figure (Figure 8) showing our proposed mechanism (see Discussion subsection “*ChrXIIr as a candidate mediator of ageing phenotypes in yeast*”).

The reviewer is absolutely correct that the association between ChrXIIr and the SEP remains correlative. We have not yet been successful in generating a stable ChrXIIr in young cells, which is what would be required to recapitulate the phenotypes of ageing in a non-aged cell and actually prove that ChrXIIr is causal. We have attempted to be very careful in our wording to not to claim proof but rather association, and cite ChrXIIr as a strong candidate for causing senescence.

At different points in the manuscript, the authors contradict themselves about whether chrXIIr is causal for aging. For example, in the introduction, they write:

"... we implicate a large fragment of chromosome XII (ChrXIIr) as the unanticipated driver of ageing phenotypes in yeast."

while in the discussion they write:

"... ChrXIIr and ERCs can only be mediators of specific pathology rather than primary drivers of ageing ...."

Unfortunately we used the word 'ageing' loosely in the second statement (which has been removed from the re-written Discussion section anyway).

In our new Discussion we make a much stronger argument that age associated phenotypes (such as the SEP) are not necessarily the limiters of lifespan, and we treat the word ageing itself very carefully as this conflates both age-associated phenotypes and lifespan. ChrXIIr accumulation is strongly associated with ageing phenotypes, but does not seem to be associated with lifespan.

We hope the Discussion is now clearer on this subtle but important point. We have tried to improve the text throughout to make it clear that our findings relate to ageing phenotypes, and particularly senescence, and we are not drawing conclusions about lifespan.

In addition to clarifying their thoughts about the role of chrXIIr in aging, the authors must perform the experiment described in point 1. Below that, I make suggestions and comments to improve this very interesting manuscript.

1.	Make a MATa/MATa diploid with the XII<>V translocation strain and compare this to a WT (no translocation) MATa/MATa diploid with regard to (a) replicative lifespan; (b) accumulation of chrXIIr during aging (MEP); and (c) appearance of Tom70 foci during aging (MEP). Transcriptomic analyses of these two strains would be nice as well, but not required.

We thank the reviewer for suggesting this – we were unaware that a:a diploids could be constructed in this way. We have replaced the original translocation experiment with an equivalent experiment using diploid cells, and the results are the same. Regarding the data specifically requested:

a) Unfortunately, we have not been able to include replicative lifespan measurements as we tried the experiment repeatedly but failed to get sensible numbers, perhaps there is something problematic about a:a diploids in this regard.
b) We have included RNA-seq to call ChrXIIr amplification as we find this to be more informative than genome resequencing – see new Figure 7C. As in the previous comparison with haploids (old Figure 5C) we see minimal age-related increase in ChrXIIr gene expression in the translocation strain.
c) We assessed Tom70-GFP intensity and again observed equivalent behaviour between wild type and translocation strains – see new Figure 7D left panel.

Though we were unable to collect the lifespan data, it is clear from the WGA signal in the imaging flow data (new Figure 7D middle panel) that the translocation strain ages at the same rate as the wild type up to 48 hours, so is clearly not short lived. However we think the reviewer is interested in whether there is a lifespan extension in this mutant and unfortunately we cannot say. We do not think this is critical for the interpretation of this experiment as we have not made any claim about lifespan, only about SEP phenotype, which we can effectively measure.

2.	The authors write: "In the XIIr <> V strain, ChrXIIr gene mRNAs did not increase in abundance with age, confirming that this effect arises through rDNA induced chromosomal instability (Figure 5E)." The translocation will also affect rDNA induced chromosomal instability, because when the right portion of chrXII is attached to chrV, it is no longer subject to being cut off of the end of the chromosome due to replication problems at the rDNA.

Apologies, that is the point we were trying to make, so we have reworded for clarity:

” The increase in ChrXIIr gene mRNAs that occurs with age in wild type was dramatically reduced in the XIIr <> V strain, which is as expected since these genes are no longer present on the ChrXIIr fragment accumulated after rDNA cleavage […]”

3.	The authors should include a statement explicitly stating what they believe the proximal cause of aging-related death is in strains that accumulate chrXIIr. Is it loss of heterozygosity (LOH)? Altered composition of the cell wall? Anaphase chromosome bridges due to incomplete replication at the rDNA?

As noted above, we have addressed this carefully in the re-written Discussion. We think that our data, and work from other labs, does not support the SEP being the cause of age-related death and so similarly we do not think that ChrXIIr causes death. Rather we see age-related phenotypes (including the SEP) as largely independent of lifespan, and conclude that another unknown mechanism normally limits lifespan. This separation of lifespan from age-related phenotypes is the focus of the accompanying manuscript by Horkai *et al.* It is possible of course that the lifespan limiting factor is ERC accumulation, but given that we can detect no effect attributable to ERCs we currently consider this unlikely.

4.	The authors should make clear that the senescence entry program (SEP) has not been established as the ultimate cause of aging-associated death. I have the impression that the authors believe that, by showing that chrXIIr is more tightly associated with the SEP than are ERCs, they have shown that chrXIIr is the "real" cause of aging, whereas ERCs are a bit of a red herring that are associated with aging but are not causal. I think that they are correct on the latter point, but it's important to keep in mind that phenotypes like accumulation of Tom70 foci could also be associated with, but not causal to, aging-related death.

We completely agree with this, and have read through the manuscript carefully to find and remove inadvertent suggestions that the SEP leads to death in normal ageing (see point 3 above). Particularly, there were points in the introduction where our wording hinted at this and that we have revised accordingly. Our findings disconnect ERC accumulation from the SEP and other ageing phenotypes, but we cannot formally exclude that ERCs impact the mechanism which causes cell death in normal ageing, and have tried to ensure that we do not make that claim.

5.	Early on, the authors should point out that they are working with diploids, and also add a comment about why they use diploids (e.g. it allows cells to remain viable even following a LOH event). An appropriate place to insert this information would be here: "To investigate the role of ERC accumulation during replicative ageing in S. cerevisiae, we examined aged mutant cells that fail to accumulate ERCs during ageing"

Yes - emphasising that cells are diploid early on would be helpful. We have added the following sentence to the second paragraph of the results section where we describe the MEP system:

"Unlike many ageing paradigms in yeast, the MEP system uses diploid cells, which are more resilient both to random mutations that could circumvent the MEP, and to toxic double strand breaks that occur increasingly with age and result (in diploids) in loss of heterozygosity (Lindstrom and Gottschling, 2009; Lindstrom et al., 2011)."

We have also included an experiment showing that Tom70-GFP and ChrXIIr accumulation also occur in haploids in Figure S4E and S4F.

6.	As I understand it, there are 187 genes (rows) in the heat map in Figure 4A. It would be helpful to mention this in the legend, since it allows the reader to associate the various numbers of genes mentioned in the text with what is shown in the figures.

Done, and for all of the hierarchical clustering analyses in the manuscript.

7.	Mention whether the following RNA is c-pro or e-pro (or neither), since these are names that some people are familiar with (I believe that it is e-pro): "In fact, the IGS1-F non-coding RNA was induced sufficiently to become the most abundant poly(A)+ RNA in aged wild-type cells (Figure S2B)."

We have added a note that the IGS1-F transcript comes from the E-pro promoter - there are two non-coding RNAs IGS1-F and IGS1-R produced from E-pro. We have also annotated E-pro and C-pro in the schematic in Figure S2A, as well as the equivalent figure in Horkai *et al.*

8.	Label the gray box on the right side of figure 2C being a key to the symbols in the main plot, since otherwise it looks like a figure in itself.

Done, thank you - we assume the reviewer meant Figure 1C, where this is indeed an issue.

**Reviewer #2:**

The authors explore the question of whether ERCs are causal in aging in budding yeast, which is a long held model in the field. The authors use the strategy of taking three yeast mutants with different levels of ERCs combined with the mother enrichment program (MEP) to see how ERCs contribute to aging. As a read out they rely on several metrics, like bud scars (WGA) to measure number of divisions, accumulation of the mitochondrial protein Tom70-GFP, and gene expression analysis. Their results are not consistent with the model. The data seems very robust with many replicates and statistics. The figures are very nice. They report on a new chromosomal element distal to the rDNA array on chr 12 that accumulates with age. They make a designer chromosome that prevents the occurrence of this species, but SEP still occurs. Their study certainly shoots a lot of holes in the model, but the new chromosomal element doesn't seem to be causal either. Although I tend to agree with the authors, based on the weight of the evidence presented, that the current ERC model is likely incorrect, the end result is somewhat unsatisfying since a new improved model doesn't really emerge, and the chromosomal species isn't demonstrated on a pulsed field gel, which seems essential. They focus the ending model on how the chromosomal species forms rather than an aging model, which is what most of the manuscript drives toward.

Yes, we agree that on re-reading the Discussion, it is too focused on the mechanism by which ChrXIIr forms and ignores the mechanism by which this could actually contribute to ageing phenotypes. We have extensively re-written the Discussion including a new schematic in Figure 8 to propose a new model for the emergence of ageing phenotypes.

Some of the main problems are

1) the pleiotropic effects the deletions will have on gene expression and cell physiology,

This was the reason we used multiple mutants that lack ERCs but are otherwise involved in different cellular processes. Effects of ERCs should be discernible at the intersect between these mutants, but actually we found essentially no overlapping effects, leading to our conclusion that the effects of ERCs are minimal.

Nonetheless we see the reviewer's point that in our set of 3 mutants the impact of individual mutations could be substantial. We have added data showing that the mutants have very different gene expression impacts in young cells (Figures S1D, S1E), and added further mutants that separate ERCs from ageing phenotypes (*mus81*Δ and *hst3*Δ *hst4*Δ, Figures 6 and S7 respectively), which completely agree with the results of the original mutant analysis, further reducing the likelihood that the effects we see result from individual mutations. Furthermore, we have included data from wild-type cells aged under different conditions, which show a range of SEP phenotypes, ChrXIIr and ERC levels (data from the Horkai et al. companion manuscript). The combined dataset now (Figure 6F,G) shows an excellent association between ChrXIIr and the SEP that is robust across growth conditions, but no association between ERCs and the SEP.

2) the heavy reliance on specific markers i.e. Tom70-GFP without explaining why this specific marker should be trusted in all mutant contexts,

We agree that more markers would be helpful as the reviewer is correct that there may be mutant-specific effects on particular markers. In addition to the Tom70-GFP and Rpl13a-mCherry markers previously presented, we have added data for nucleolar RNA polymerase I marker Rpa190-GFP and vacuolar marker Vph1-mCherry to complement the Tom70-GFP data, together covering ageing phenotypes for different cellular functions described by multiple labs (characterised in PMIDs: 24332850, 32675375, 23172144, 31291577). Additional markers are shown in Figures S3E and S3F and corroborate the Tom70-GFP findings in Figure 3A, i.e. that ERC accumulation is not consistently associated with any specific ageing marker.

3) the complexity of the multiple gene expression models which are difficult to understand,

The model that we apply to deconvolve the effects of ERCs and the SEP from age and genotype is just an extended variant of the standard DEseq2 pipeline. However, we agree it was not well explained, so we have carefully rewritten the text where we introduce this model (Figure 2) to improve clarity, and made it clearer later in the text where we look for gene expression changes linked to the SEP (Figure 4) that we are using exactly the same modelling approach. We have also highlighted where we are talking about ERCs and Age specifically as variables in the model by underlining all model variables where discussed as it was not always obvious to what we were referring.

4) the high demand on the reader to keep track of many correlating phenotypes and for relevant background knowledge,

We agree, but the useful outcomes of our work arise only through intersecting many different approaches and mutants. We have extensively revised the text to improve clarity and hope that the approaches are now more coherent and easier to follow.

5) the lack of a new model to explain aging,

As noted above, we agree that on re-reading the discussion it is too focused on the mechanism by which ChrXIIr forms and ignores the mechanism by which this actually contributes to ageing phenotypes. We have put a new section at the start of the Discussion with a new schematic in Figure 8 to specifically address this point (see Discussion subsection “*ChrXIIr as a candidate mediator of ageing phenotypes in yeast*”).

5) the lack of evidence for the chromosomal species. The title implies "chromosome XII cleavage" is critical for senescence which doesn't seem appropriate since cleavage itself isn't measured, how the species is generated is only a model, and a strain that doesn't generate the species still shows senescence according to Tom70-GFP.

The ChrXIIr species was demonstrated to exist as a separate fragment by PFGE in the Hu *et al.* publication where it was first described (Figure 6D in that paper, PMID: 24532716, it is the heterogeneous signal in the aged sample with a minimum size of 0.6Mb). Notably, this study was performed in the same MEP wild-type strain that we are using so the result should be fully applicable to our system. We have now made this clear in the text:

” The increased expression of genes on ChrXIIr with the SEP strongly suggested the accumulation of a chromosomal fragment from this region; indeed a discrete chromosomal fragment arising through rDNA cleavage and containing part of the rDNA along with the chromosomal region centromere distal to the rDNA has been previously described in aged wild-type MEP cells and validated by pulsed field gel electrophoresis (Hu et al., 2014; Pal et al., 2018).”

We agree that the title was slightly misleading and have modified it to focus on the fragments (which we can detect) rather than the cleavage events (which remain hypothesised).

We have addressed the issue of why the ChrXII translation strain still undergoes the SEP in the new Discussion section, and improved the clarity of the Results section where this strain was introduced (Results subsection “*Amplification of protein coding genes on ChrXIIr does not cause senescence*” and new Figure 7). Although the translocation removes the ChrXIIr genes (and about 0.6 Mb of DNA), cleavage of the rDNA will still generate chromosomal fragments up to ~1.8 Mb in size, depending where in the rDNA the cleavage occurs (illustrated in the Figure 7A schematic). The translocation strain simply shows that copy number amplification of the genes on ChrXIIr is not responsible for the SEP, implicating instead the large linear DNA fragment itself rather than any particular gene. We have redrawn the schematic figures for this experiment to scale (Figure 7A), which makes the relative sizes of the rDNA and genic regions of ChrXIIr much clearer.

I have made specific suggestions below for improvement. However, the resolution of these issues may be insufficient to justify publication since some of my key problems relate to the larger issue of the story the authors are trying to tell with the data that they currently have.

Specific suggestions

1.	I suggest adding a small annotation to each mutant to indicate ERC hi or ERC low so that reader does not have to keep referring back to figure S1. ERCs in the mutant strains should be quantified as in S1A. In some of the figures it might help to plot the lack of correlation between ERCs and the phenotype being described.

Apologies - we have not communicated the experimental logic well enough here. All the mutants (*rad52*Δ, *spt3*Δ and *sac3*Δ) are selected for having dramatically reduced levels of ERCs (5x-30x). There is no different interpretation between these mutants and they are all treated as 'ERC low' in the model. We have emphasised this point and added quantification data to the southern blots in Figure S1A and S1B as the reviewer suggests. To clearly articulate the experimental design, we have added the following paragraph to the end of the first results section:

” Together *rad52*Δ, *spt3*Δ and *sac3*Δ form a set of three mutants that accumulate very few ERCs during ageing compared to wild type. Consistent differences that emerge during ageing between each mutant and the wild-type form candidate effects that could be attributable to ERCs, whereas differences observed in only one mutant or that are already present in young cells prior to ERC accumulation must be attributed to genotype effects rather than ERCs. We note that *rad52*Δ and *sac3*Δ are short lived whereas the lifespan of *spt3*Δ is almost identical to wild type, providing a spectrum of lifespans to allow separation of terminal phenotypes from effects of ERCs (Lim et al., 2021; Lim et al., 2022; Park et al., 1999).”

In addition, we note that the mutants added to the revised manuscript (*mus81*Δ and *hst3*Δ *hst4*Δ) are also low-ERC mutants. Southern blots and quantifications for these are present in Figure 6A and S7C respectively.

The correlation plots are a good idea – we have added such plots in Figure 6F,G showing the strong correlation between ChrXIIr and Tom70-GFP and poor correlation between ERCs and Tom70-GFP in individual wild-type replicates using the large dataset of matched RNA-seq and flow cytometry samples from Horkai *et al*.

2.	If there were other SAGA or TREX mutant have accumulate ERCs to a high level, and their gene expression profiles were similar to spt3 or sac3, this would strengthen the gene expression argument.

We agree that it would be ideal if we could make this comparison, however all the tested SAGA and TREX mutants impair ERC accumulation, so we do not know of any mutants that fit this description. Nonetheless, it is an important point that we did not include validation data to show just how different the effects of each mutant are; we see how in the absence of this data it would be easy to assume that *spt3*Δ and *sac3*Δ mutants have similar effects on gene expression given that they affect a pair of physically interacting complexes. In reality however, the effects of these mutants on gene expression are almost diametrically opposite, and are both very different from *rad52*Δ (see new Figures S1D and S1E). The following additional text and associated figure panels address this point:

“Our strategy of defining the effects of ERCs during ageing based on similar gene expression changes in otherwise disparate mutants that all lack ERCs (spt3Δ, sac3Δ and rad52Δ) relies on these mutations having different effects on gene expression in young cells. In support of this, Principal Component Analysis (PCA) performed on the log phase samples shows no association between any pair of mutants (Figure S1D). In more detail, pairwise differential expression analysis at log phase uncovered >1,000 significantly differentially expressed genes in each mutant compared to the wild type (DESeq2, Benjamini-Hochberg (BH) corrected false discovery rate (FDR) < 0.01, log2-fold change threshold ± 0.5), and of the 2606 genes that are significantly differentially expressed in at least one mutant only 197 (7.5%) change in the same direction in all 3 mutants (93 up, 104 down). Hierarchical clustering of the 2606 genes also shows little overlap between the effects of the different mutants (Figure S1E). Notably, spt3Δ and sac3Δ, which might be expected to have similar transcriptomic effects given that Spt3 and Sac3 are physically associated and link promoters to the nuclear pore, actually have almost diametrically opposite effects on gene expression.”

3.	In figure 2B it makes more sense to me to put the spt3 and sac3 profiles together.

We agree and have rearranged all plots across the manuscript to put *spt3*Δ and *sac3*Δ together.

4.	The authors use mutations that disrupt gene expression due to effects on RNAPII. How do we know that the Tom70 phenotype cannot be attributed to different effects in each mutant?

We understand this concern and should have included better validation data showing that the mutants have very different effects on gene expression – this data has now been added, see response to point 2 above. Concerning the specific effects on Tom70-GFP, we find 187 genes that tightly associate with the Tom70-GFP phenotype across the data set (Figure 4A). If the Tom70-GFP phenotype was caused by specific gene expression differences resulting directly from the mutations, we would expect those differences to be already extant before ageing. The vast majority of genes associated with the Tom70 phenotype follow the expression pattern *rad52*Δ > wild type > *spt3*Δ > *sac3*Δ in aged cells (Figure 4A). However, at log phase, only 4 genes out of 2606 that are significantly affected by at least one mutant follow the pattern of being higher than wild type in *rad52*Δ but lower in *spt3*Δ and *sac3*Δ. Therefore it is not possible to explain the associations we draw between senescence and gene expression by impacts of the individual mutations on gene expression alone.

We also note that the addition of two other ageing markers for different organelles (Rpa190-GFP and Vph1-mCherry) further reduces the likelihood that the phenotypes we see result from individual gene expression effects of the different mutants (Figure S3E, S3F) (see point 5 below), as does the addition of further mutants *mus81*Δ and *hst3*Δ *hst4*Δ (Figures 6, S7), which also follow the patterns we describe.

5.	The heavy reliance on Tom70-GFP as a metric for SEP needs to be justified. In fact in Horkai et al the introduction mentions a trajectory that is not associated with Tom70 accumulation.

To address this point we have added data for the vacuolar marker Vph1-mCherry and the nucleolar RNA polymerase I marker Rpa190-GFP. These have been validated to change in specific ways during ageing (PMIDs: 31291577, 25190112), and show the same behaviour in *rad52*Δ and *spt3*Δ mutants with dramatically reduced ERC burdens (Figures S3E, S3F). Therefore just as for Tom70-GFP, we must conclude that these age-associated changes are not caused by ERCs. The following new text addresses this point:

To confirm that ageing phenotypes are not connected with ERC accumulation we examined other previously described markers. Firstly, the size of the nucleolus, visualised as the area occupied by the RNA polymerase I marker Rpa190-GFP, has been shown to expand as cells age (Morlot et al., 2019). This is thought to result from increased rDNA copies arising through “ERC amplification occupying more space and recruiting more ribosome biogenesis factors, however we observe that this increase of nucleolar size with age occurs in wild type and in both rad52Δ and spt3Δ mutants that accumulate ≥10-fold less ERCs (Figure S3E). Secondly, the vacuole expands with age and increases in pH resulting in a higher signal from a Vph1-mCherry reporter (Hughes and Gottschling, 2012). Again, we observe the same phenotype in wild-type and mutant cells, except that the increase stops at 24 hours in the rad52Δ mutant as these cells have already completed their replicative lifespan (Figure S3F). Our imaging data for Tom70-GFP, Vph1-mCherry and Rpa190-GFP, as well as direct measurements of growth rate and gene expression dysregulation all lead to the same conclusion: that age-associated changes and the onset of senescence marked by the SEP occur independent of ERCs.

Yes, there is an ageing trajectory not associated with Tom70-GFP accumulation, but senescence (the slowing down of the cell cycle with age) is almost absent on this trajectory, consistent with the suggestion that Tom70-GFP is a valid SEP marker (PMID: 32675375).

6.	The explanation of the gene expression analysis in the last paragraph on page 5 is difficult to understand.

We agree and have simplified this paragraph, which describes why we consider that the gene expression differences identified by the first linear model as resulting from ERCs are likely to stem from genotype specific effects.

7.	How well accepted is gene expression as a metric of aging?

Progressive gene expression changes during yeast ageing have been widely reported (PMIDs: 30274593, 24532716,30334737,14718559,26422514,18245757 and others), more so indeed than any individual fluorescent marker to our knowledge. We consider this reproducibility makes gene expression a good marker in itself. However, a major reason for using gene expression was as an orthogonal marker to fluorescent reporters, which individually can be prone to artefacts. We consider that the combination of multiple fluorescent markers with gene expression data that we now employ in both this manuscript and Horkai *et al*. represents an exceptionally robust set of ageing data.

8.	The authors use "non-ChrXIIr genes" as a control in several experiments, but it would be important to report also on using the rest of ChrXII as a control, since using the entire genome could easily wipe out non-euploid metrics.

We agree, and have added the data for the rest of ChrXII to Figure 5C (the genome resequencing results), the result is similar to the non-ChrXII genes. We thank the reviewer for suggesting this as it motivated us to look more closely at individual chromosomes – we do indeed observe other aneuploidies emerging albeit to a much lesser extent than ChrXIIr, and added this data as Figure S6.

9.	The authors refer to ChrXIIr as extrachromosomal, but I don't see evidence to support this claim. The fragment could remain connected to ChrXII.

And also 10.	The fragment needs to be visualized on a PFG.

The ChrXIIr species was demonstrated to exist as a separate fragment by PFGE in the Hu *et al.* publication where it was first described (Figure 6D in that paper, PMID: 24532716, it is the heterogeneous signal in the aged sample with a minimum size of 0.6Mb), using the same MEP strain as we have used for this study. We have now made this clear in the text

“The increased expression of genes on ChrXIIr with the SEP strongly suggested the accumulation of a chromosomal fragment from this region; indeed a discrete chromosomal fragment arising through rDNA cleavage and containing part of the rDNA along with the chromosomal region centromere distal to the rDNA has been previously described in aged wild-type MEP cells and validated by pulsed field gel electrophoresis (Hu et al., 2014; Pal et al., 2018).”

11.	As the amplification of ChrXIIr does not occur in the engineered strain (Fig 5) but the SEP still occurs based on Tom70, how can the authors conclude the region is associated with SEP as marked by Tom70-GFP??? What aspects of the expression profile persist with the engineered chromosome? This engineered chromosome is a key reagent to the story.

Apologies, this was poorly explained. We have addressed the issue of why the ChrXII translocation strain still undergoes the SEP in the new Discussion section. Although this translocation removes the ChrXIIr genes (and about 0.6 Mb of DNA), cleavage of the rDNA will still generate chromosomal fragments up to ~1.8 Mb in size, depending where in the rDNA the cleavage occurs (see schematic Figure 7A). The translocation strain simply shows that copy number amplification of the genes on ChrXIIr is not responsible for the SEP, implicating instead the large linear DNA fragment itself rather than any particular gene. We hope the better schematic drawings we have added to Figure 7 that show the rDNA to scale with the rest of Chromosome XII make this clearer.

A few typos:

Figure 2D legend does not mention the cell clusters Figure 6 labels, "specific" not secific Figure 2, S2 title, expression change not charge

Thanks - corrected.

**Reviewer #3:**

In the manuscript "Senescence in yeast is associated with chromosome XII cleavage rather than ribosomal DNA circle accumulation" Zylstra et al. challenge the widely accepted view that extrachromosomal rDNA circles (ERCs) are major contributors to cellular senescence and propose instead that accumulation of a fragment of chromosome XII is strongly associated with aging. This conclusion is based on the analysis of purified populations of aged mother cells of different mutant strains that accumulate less or no ERCs. In these populations, the authors perform transcriptome analysis and correlate it with different markers of cell senescence, such as the accumulation of the outer mitochondrial protein Tom70 or cell size.

In my view, the evidence suggesting that ERCs play only a minor role in cell senescence is not conclusive and not sufficient to refute previous evidence showing a strong association between the senescence transition and ERC accumulation, in particular from recent single cell-based studies (PMID: 31291577, PMID: 30042134, PMID: 32675375).

Yes, we have thought a lot about these studies suggesting that ERCs cause the SEP. We already noted the first two as primary evidence for the contribution of ERCs to senescence in the introduction, but we agree it is useful to specifically compare and contrast these with our findings and we have now done so in the Discussion. Particularly see the second paragraph of subsection “*ChrXIIr accumulation can substitute for ERCs in many models of ageing*” where we directly address the three publications the reviewer highlights, but here are more in-depth discussions:

In 31291577 (Morlot *et al*), ERC accumulation was measured using a copy number sensitive rDNA reporter to show that the timing of ERC accumulation above background always rapidly precedes SEP onset. However, our data (now expanded) shows beyond reasonable doubt that the SEP also occurs in strains that have far less or no ERCs, so ERCs cannot be causal for the SEP. We think that the association between ERCs and the SEP reported by Morlot et al. stems from the unknown underlying process that increases the risk of replication-related rDNA damage per division with age (Lindstrom et al. PMID: 21436897). Both ERC formation and ChrXIIr formation are independent outcomes that can arise through rDNA replication stress, so in every cell, as the risk of rDNA replication defects rise with age, the risk of ERC formation (leading to progressive, probably exponential, accumulation) and the risk of ChrXIIr formation (leading to the SEP) both increase. We think that ERC formation is probably the more likely outcome for any given rDNA damage event and hence cells will tend to have formed a number of ERCs prior to ChrXIIr formation and consequent SEP. However, ERC accumulation to a detectable level takes multiple cell divisions so although on average ERC accumulation and the SEP may appear to co-occur, the relative timing will be very variable across individual cells.

Inspection of the data presented by Morlot et al shows exactly this. The single cell trajectories of nucleolar GFP-LacI fluorescence in Figure 1D shows substantial variation in the timing of ERC accumulation relative to the SEP, with ERC accumulation occurring long before or long after the SEP in many cells. Conclusions are drawn from the averaged data in Figure 1E plotted with standard errors (which quantify error in the mean) as opposed to standard deviation (which quantifies the spread in the population); this is a valid approach given the difficulties and associated uncertainties in the measurements, but also removes deviations from average behaviour across the population. Therefore, we do not think that the data presented by Morlot et al demonstrates that ERCs are the cause of the SEP, just that they are on average temporally associated with the process that causes the SEP, and that is also consistent with our ChrXIIr model.

In 30042134 (Neurohr et al.) an rDNA-binding fluorescent marker is used to visualise ERCs, and the senescence phenotype is shown to transfer to daughter cells along with ERCs during occasional transfer events. However, our results indicate that the mother cell retention mechanism of ChrXIIr is probably the same as for ERCs and therefore ChrXIIr would be transferred to the daughter with the ERCs. Indeed, the Cfi1 marker used in this study would not distinguish ChrXIIr from ERCs. Again, this paper does not counter our primary point - we observe robust senescence in multiple mutants with severely curtailed ERC accumulation and (in Horkai *et al.*) little senescence in wild-type cells that accumulate high levels of ERCs, so the SEP cannot be attributed to ERC accumulation.

32675375 (Li et al.) did not directly address ERCs, but examines nucleolar expansion and fragmentation which is thought to result from ERC accumulation. However, we now show that nucleolar expansion occurs independently of ERCs (Figure S3E) so this cannot be taken as evidence of a contribution of ERCs to senescence. It is also worth noting that in Li et al. the ageing trajectory that involved nucleolar fragmentation was the low senescence trajectory, so even if nucleolar fragmentation is indicative of ERC accumulation, this cannot have caused the observed senescence.

The observation that a part of chromosome XII (XIIr) accumulates in old cells is interesting but not per se novel (PMID: 24532716), which the authors acknowledge, but whether and how XIIr accumulation contributes to aging has not been clear. The authors have developed an elegant tool to investigate the functional consequence of XIIr accumulation using chromosome engineering. However, preventing accumulation of XIIr does not alter the investigated aging hallmarks, and is thus rather a consequence than a driving force of aging.

We have addressed the issue of why the ChrXII translocation strain still undergoes the SEP in the new Discussion section. ChrXIIr has two parts: 0.6 Mb of DNA containing ~330 genes, and 0-1.8 Mb of rDNA, the size of which depends on where the cleavage event occurs with the rDNA. Although the translocation we introduced removes the ChrXIIr genes (and about 0.6 Mb of DNA), the mechanism that forms ChrXIIr will still form a large linear fragment containing up to 1.8 Mb of rDNA. The translocation strain simply tests whether copy number amplification of the genes on ChrXIIr is responsible for the SEP, which it isn't, but we do not have a means to prevent formation of the large rDNA containing part of ChrXIIr except indirectly using mutants or different medias, for which we now provide additional data in Figures 4 and 6. We have also redrawn the schematic figure for the translocation experiment to scale (Figure 7 A and B), which makes the relative sizes of the rDNA and genic regions of ChrXIIr much clearer.

This leaves the interesting question of why accumulation of this chromosomal fragment is so tightly associated with aging cells. I believe that especially the role of this recurrent chromosomal abnormality is interesting to the PLOS Biology readership, but I have a number of concerns, some of which I also outlined in detail in the review to the accompanying manuscript "Dietary change without caloric restriction maintains a youthful profile in ageing yeast" from the same group. The first point applies to both manuscripts and parts of it are a direct copy.

1)	My main concern is the interpretation of the transcriptome data. The indicated age dependent decrease in ribosome biogenesis genes is reminiscent of activation of the environmental stress response (ESR), which is a hallmark of aging, slow growth or permanent cell cycle arrest (PMID: 22498653, PMID: 17105650, PMID: 30739799). A terminal cell cycle arrest is entered at the end of replicative life irrespective of why and how fast cells aged. The fraction of terminally arrested cells in each population of purified aged mother cells could thus dominate the transcriptome. rad52∆ mutant cells for example have a much shorter RLS than wild type cells (11 vs 25-30, (Delaney et al 2013)). That means that at the 24h timepoint, all rad52∆ cells are terminally arrested while the wild type cells are still all cycling, while at the 48h timepoint probably most cells have entered a terminal cell cycle arrest.

If the gene expression profile in aged populations is a consequence of arrested cells, it changes the interpretation of the transcriptome data. For example: If rad52∆, spt3∆ and sac3∆ mutants all display similar age-dependent gene expression changes after 48h of aging as wild type cells, this could mean that by that point they have mostly entered a terminal cell cycle arrest and not that they all age in the same way.

Such data can therefore not be used to rule out the contribution of ERCs to aging in wild type cells. The authors need to control their transcriptome data for this cell cycle arrest effect by A) determining division times and the fraction of terminally arrested cells in the aged populations and B) comparing what part of the gene expression change is driven by activation of an ESR, for example by comparing to published data sets of slow growing or arrested cells.

We completely agree that growth rate is a major contributor for many of the gene expression changes during ageing that we and others report, however our results show that terminal arrest cannot be the primary driver. We demonstrate in Horkai *et al.* that age-linked gene expression changes occur during ageing on glucose but far less so on galactose, despite the proportion of terminally arrested cells being the same or greater in galactose at all time points (see figures 1D and 2A of Horkai *et al.*). Instead, we see an excellent correlation between the SEP marked by Tom70-GFP and gene expression change. As the reviewer notes, gene expression is strongly influenced by growth, so the dramatic slowing of the cell cycle after the SEP would be expected to be a major contributor to observed age-linked gene expression change.

As the reviewer suggests, we have deconvolved the impact of senescence on the transcriptome, through an adjustment of our linear model design. Senescence is indeed the major contributor to gene expression change during ageing in glucose, causing the global changes in gene expression pattern (Figures 4E, 4F). As shown in the new Figure S5, the impact of senescence is indeed similar to the ESR for ESR genes, whereas the gene expression impact that the model attributes to age *per se* is not similar to the ESR. The impact of senescence also parallels the impact of slow growth as the reviewer predicts, based on the Regenberg *et al.* dataset. However, we find additional gene expression signatures in the SEP that are not observed under stress or slow growth.

We have added to the revised Discussion the idea that much of the gene expression effect attributed to ageing actually therefore arises as a normal cellular response to the slowing of growth after the SEP. This fits well with our observation that cells which do not undergo senescence (marked by Tom70-GFP, and now also validated by growth measurements in Horkai *et al.* Figure 1F) reach terminal arrest without major changes in gene expression. Once at terminal arrest, cells are generally considered ‘dead’ and therefore unlikely to dynamically change gene expression programme. We note that although Neurohr *et al*. measured a gene expression response to growth arrest, this was a reversible arrest using *cdc28-13* not a terminal arrest as occurs at the end of replicative lifespan, and we are not aware of any evidence that the terminal arrest at the end of life is associated with a gene expression change. Indeed Neurohr *et al.* specifically attributed the gene expression change in this arrest system to continued cell growth (in terms of biomass) without cell division. In contrast, *rad52*Δ and *hst3*Δ *hst4*Δ mutants which show complete terminal arrest by 24 hours do not not show any increase in cell size from 24 to 48 hours nor any change in gene expression pattern (Figures 2C, 7B). However, the reviewer is correct that gene expression changes which our linear models attribute to Age arise from a direct comparison of log phase cells which are not arrested with 48-hour aged cells that are mostly terminally arrested and we cannot exclude a contribution from the terminal arrest; we have now noted this in the manuscript.

2)	Because of the complications described above, a rad52∆ mutant (and other DNA damage repair mutants) is a particularly poor model to study aging because cells stop dividing after roughly 10 divisions and mostly arrest as budded cells (indicative of G2/M arrest potentially linked to persistant DNA damage), in contrast to wild type cells that primarily arrest in G1 after 20-30 divisions (Delaney et al 2013). Despite the problematic use of rad52∆ as aging model, several interpretations strongly depend on the rad52∆ mutant strain, which often shows stronger and opposite phenotypes compared to spt3∆ and sac3∆. For example, accumulation of Tom70-GFP is accelerated in rad52∆ but occurs more slowly in spt3∆ and sac3∆ leading the authors to conclude that ERC accumulation and senescence transition are not related, because all of the above strains have no or reduced levels of ERCs. However, if the rad52∆ data were excluded from this analysis, ERC accumulation and Tom70-GFP accumulation would correlate. Similarly, age dependent changes in gene expression (Figure 2B, 2D, 4A) are often going in opposite directions in rad52∆ than in spt3∆ and sac3∆ mutants. While these data do not necessarily support a role of ERCs in affecting gene expression or the senescence transition, I don't think they present evidence against it either. Because of the above-mentioned issues with the rad52∆ mutant, I am very skeptical about results that are solely supported by the rad52∆ results.

We thank the reviewer for pointing this out - we checked and our *rad52*Δ mutant does indeed arrest 80% in G2 / S (this can be assayed in imaging flow cytometry based on budded versus unbudded cells, Figure S7A). We note that our imaging flow cytometry data for Tom70-GFP and all other markers is filtered to include only G1 cells based on circularity so the Tom70-GFP data is comparable between the strains - we have emphasised this point in the manuscript. The sequencing data is derived from the whole population however, so for this the reviewer's point is valid.

We have therefore looked for other mutants that have reduced ERCs and show comparable gene expression phenotypes to *rad52*Δ during ageing, but are more similar to wild type. This is not trivial - mutants in ERC retention also impact ChrXIIr and hence also reduce senescence so we have to look at mutants in ERC production, which are universally short lived apart from *fob1*Δ which as we show in Figure S1A has no effect on ERCs in aged MEPs.

We have found two informative mutants and added these in Figures 6 and S7 to provide further validation. Firstly, *mus81*Δ is particularly useful as it has a 10-fold reduction in ERCs but only mild ageing and cell cycle differences compared to wild type, and does not share the early strong SEP phenotype of *rad52*Δ. As predicted from our analysis, *mus81*Δ cells are very similar to wild type in Tom70-GFP, gene expression change and ChrXIIr accumulation. Secondly, *hst3*Δ *hst4*Δ mutants die young and have a strong early SEP phenotype just like *rad52*Δ, but have only a mild reduction in ERCs and show less bias towards a G2 terminal phenotype. Nonetheless, they display the same Tom70-GFP, ChrXIIr accumulation and gene expression phenotypes as *rad52*Δ mutants, disconnecting the strong SEP phenotype observed in *rad52*Δ cells from the strong G2 bias or the unusually low ERC levels.

Finally, we have tested the association between ChrXIIr, ERCs and the SEP across many wild-types aged under different conditions, and again observe an excellent association with ChrXIIr but no correlation with ERCs (Figure 6F,G).

Overall, although we agree that *rad52*Δ is a very unusual mutant, we have been able to validate the observations in this mutant using other mutants and growth conditions, meaning that the lack of association between ERCs and the SEP cannot be attributed simply to peculiarities of this mutant.

3)	Li et al 2020 (PMID: 32675375) have shown that budding yeast cells can follow different trajectories during replicative aging and identified at least two different modes characterized by either accumulation of nucleolar markers (indicative of ERC accumulation) or accumulation of aggregated mitochondria. Cells that loose mitochondrial function tend to slow down cell divisions earlier and stop dividing earlier. These aging trajectories are influenced by genotype, in particular mutant cells that prevent ERC accumulation follow the "mitochondria" aging trajectory. Thus, while Tom70-GFP may be a good marker for SEP in some cells, this may not be the case for all aging cells. To directly measure SEP, the authors should determine cell cycle durations in populations of aged cells.

Firstly, we see the Li et al paper as excellent evidence that ERCs are not the cause of senescence, since the authors observe nucleolar fragmentation and loss of rDNA silencing that they attribute to rDNA instability in the low senescence trajectory, whereas the high senescence trajectory does not show this. However, we accept the reviewer's point here that Tom70-GFP may not be a universally applicable SEP marker. We have therefore added data for the vacuolar marker Vph1-mCherry and the nucleolar RNA polymerase I marker Rpa190-GFP. These have been validated to change in specific ways during ageing (PMIDs: 31291577, 25190112), and follow the same course in *rad52*Δ and *spt3*Δ mutants with dramatically reduced ERC burdens (Figures S3E, S3F).

Direct measurement of cell cycle during ageing for these mutants is problematic - experiments in collaboration with the Charvin lab revealed that both *spt3*Δ and *rad52*Δ cells rapidly formed aggregates during ageing in microfluidic devices that prevented a useful analysis. However we agree it is vital to understand whether the Tom70-GFP accumulation observed in a low ERC mutant is actually associated with a growth defect. We therefore isolated live cells from aged wild type and *spt3*Δ MEP cultures and measured the time required for these to form colonies. Colony formation was dramatically retarded in both aged wild type and aged *spt3*Δ (Figure S3D), consistent with a profound delay in cell cycle. Notably an equivalent experiment using galactose aged cells that show very low Tom70-GFP during ageing did not detect a significant difference in colony formation between young and aged cells (see new Horkai *et al.* manuscript Figure 1F).

4)	The observed accumulation of a fragment of Chromosome XII is interesting. In particular, the XIIr <> V chromosome engineering experiment is very elegant and demonstrates that XIIr accumulation does not cause SEP itself. It would be interesting to further investigate the role of this chromosome fragment: If it is acentromeric, as suggested by the authors, do multiple copies accumulate in old mother cells (could be analyzed in single cells using FISH)? If not, is chromosome XII fragmentation a terminal event in replicative aging? An interesting idea would be that one cause of cell death in aged cells are irreparable recombination intermediates in the rDNA. This could be the reason why a fraction of old cells (30% in wt, 70% in rad52∆) arrest as budded cells and would be compatible with the observation that swaping the telomere proximal region of Chromosome XII had no effect on aging. It would predict that the telomere proximal region of Chromosome V is not associated with aging.

Yes, we agree this is a very interesting area. We considered carefully the idea that an unresolved replication intermediate may underlie the terminal arrest, at least in a fraction of cells, but cannot see how an unresolved replication intermediate could form the copy number amplification we observe by RNA-seq and genome re-sequencing without a mitosis event. In theory Break Induced Replication (BIR) could re-replicate a region the size of ChrXIIr, but BIR requires Rad52 yet ChrXIIr accumulation is greatest in *rad52*Δ mutants. These considerations led to the model we propose in Figure 9 in which it is the resolution of unresolved replication intermediates during mitosis that forms ChrXIIr.

Regarding copy number, ChrXIIr does accumulate but not very much, only reaching at most an average of 1 copy per cell. Given that the vast majority of cells in populations with high ChrXIIr (eg: wild type 48h or *rad52*Δ 24h) are past the SEP (whether they have reached terminal arrest or not) and ChrXIIr is very tightly associated with the SEP, it seems unlikely that many cells would have more than 1 copy of ChrXIIr as that would also mean that many post-SEP cells have no copies. Therefore, although ChrXIIr presumably replicates in S phase this does not seem to increase the copy number through the ~2-4 cell divisions that occur post-SEP. Where does it go? It is possible that asymmetric segregation is less efficient for ChrXIIr than for ERCs so that ChrXIIr copies are partitioned to daughter cells in many cases. Alternatively, during G1 arrest the ChrXIIr fragment could be degraded, and this degradation event may even be the cause of the G1 arrest. However, we are not convinced of either of these explanations, and while we agree that this is a fascinating question, it is one that we will need to explore in depth and aim to do so in a future study.

Reviewer #4:

In this manuscript, Zylstra et al studied yeast aging in the presence/absence of ERCs, and found that accumulation of ChrXIIr (instead of ERCs) was the primary driver of yeast aging. As their markers, the authors use Tom70-GFP showing the onset of cell division defects at the SEP, and gene expression differences/similarities measured by RNAseq in different genetic backgrounds allowing/blocking formation or accumulation of ERCs.

This is a provocative manuscript with novel claims. If validated by further experiments/controls, this work would mean a paradigm shift in the yeast aging field and would make the yeast model of aging more relevant to aging in higher eukaryotes. Despite all these (potential) excitement, I have some major concerns about this work and its claimed take-home messages:

1. At no point, the authors measure the replicative lifespan of the constructed strains. I would recommend some single-cell microfluidic assays to measure the lifespan effects of key strains/conditions shown in this manuscript.

Apologies- we should have made this clear. The replicative lifespans of *rad52*Δ, *sac3*Δ and *spt3*Δ mutants on YPD are known, and we have now specifically noted this in the description of the mutants at the start of the results section.

"We note that *rad52*Δ and *sac3*Δ are short lived whereas the lifespan of *spt3*Δ is almost identical to wild type, providing a spectrum of lifespans that should allow deconvolution of terminal phenotypes from effects of ERCs (Lim et al., 2021; Lim et al., 2022; Park et al., 1999)."

Although microfluidic measurements would indeed be helpful, preliminary experiments in collaboration with the Charvin lab have revealed that key mutants *spt3*Δ and *rad52*Δ are not amenable to microfluidics. In these mutants, the daughters don't detach from the mother and cells quickly and form clumps that make any tracking of the mother, or proper separation of the daughters, impossible.

2. Almost all of the claims/conclusions are based on RNA-seq-measured small expression variations. It's OK to perform this bulk/high-throughput analysis first but it would be necessary to focus on key genes later one at a time at the protein level (promoter-GFP fusions perhaps). But which genes? That's one of the problematic areas of this manuscript, as the authors looked into the pairwise expression variations (the right thing to do) but did not see sufficiently high number of genes for downstream analysis. Perhaps not surprisingly, there is no specific gene(s) mentioned/claimed as the causal contributor to the shown effects. I was not very excited about their using small expression variations to reach the provocative conclusions of this work.

We understand and share the reviewer’s frustration on this point. Given the major role in ageing ascribed to ERCs, we expected to find large, well-defined gene expression changes in their absence, but it is a key message of this manuscript that there are none!

Similarly, we hoped to find individual causative genes, but the data does not support the existence of such genes. Rather, we think that ChrXIIr accumulation generally hampers nuclear pore complex function and nuclear membrane structure (see the re-written Discussion), and this would not lead to any particularly causative genes changing in expression level. We have considered tagging ChrXIIr itself, but having dicussed this with scientists working in the field we consider it beyond the limits of current technology to detect a 1.5x shift in copy number of a single copy genomic region in live cells.

We agree that using a few small gene expression changes to support a conclusion would be risky, and also that the conclusions we draw are provocative. However, the effects we see are not small – often 4-fold or more across age – and occur across hundreds of genes. Although we do not identify particular strong effect genes, we consider that large reproducible changes in expression of a very large number of genes does constitute strong evidence. Furthermore, our most provocative conclusion comes from Figures 1 and 2 in which we show that ERCs have no major impact on the transcriptome, and we can very confidently draw this conclusion from our data.

3. Related to #2 above: Instead of small gene expression differences that are at the correlative/bulk level or SEP as the marker, there are some well-established yeast aging markers showing the pathology/physiology of yeast during its aging. It is absolutely necessary to look at least 2 such markers in the various strains the authors constructed. Equally importantly, the authors should perform live-cell tracking of aging yeast cells (carrying those markers one at a time) under the microscope and show how age affects their potential change in ERC-producing/absent/nonaccummulating cells. The same experiment should also be performed for the haploid strain which carries ChrXIIr on Chr5.

We agree that adding additional age markers is useful and important, and so have added data for the vacuolar marker Vph1-mCherry and the nucleolar RNA polymerase I marker Rpa190-GFP, which fully supports our Tom70-GFP data. These have been validated to change in specific ways during ageing (PMIDs: 31291577, 25190112), and show the same behaviour in *rad52*Δ and *spt3*Δ mutants with dramatically reduced ERC burdens as they do in wild type (Figures S3E, S3F). Therefore just as for Tom70-GFP, we must conclude that these age-associated changes are not caused by ERCs.

Unfortunately, as noted in response to point 1 above, our trial microfluidics work in collaboration with the Charvin lab revealed that *spt3*Δ and *rad52*Δ are not amenable to microfluidics. However, we dispute the assertion that live cell imaging data is required to support our claims. Our imaging data shows that age-linked changes in Tom70-GFP, Vph1-mCherry and Rpa190-GFP that have been previously been attributed to ERC accumulation still occur in strains lacking ERCs. Even if live cell imaging showed that the dynamics of these markers are slightly different in the absence of ERCs that would not alter our conclusion that these changes are not caused by ERC accumulation. The same argument applies to the translocation strain: Tom70-GFP intensity and gene expression change in the same manner irrespective of the translocation, so these phenotypes cannot be attributed to specific genes on ChrXIIr.

4. Studies aiming to track rDNA copy-number or its 3D dynamics are hard to perform due to genomic integration hardships (in a precise/countable manner) associated with its multi-copy nature. However, on the initially-nonrepetitive ChrXIIr, the authors can easily integrate a strong promoter driving a fluorescent reporter to the ChrXIIr region and see in live/aging cells how its expression (as a proxy for copy number changes) changes by replicative age. LacO arrays with LacI could also be integrated to observe the translocation dynamics of this region but perhaps that would be the aim of another paper.

As noted above, we would love to do this experiment, and it would address the reviewer’s valid concern raised in point 2. However, we are not confident that a fluorescent reporter would work as so many indirect changes to gene expression occur during ageing - we have not for example identified a strong promoter that we are confident maintains constant expression with age to a sufficient extent to detect a 1.5-fold difference. We therefore think that the LacO/LacI approach suggested by the reviewer would be required, but the technical difficulties here are formidable and agree this will need to be the focus of another paper.
