# Supplementary material for: Senescence in yeast is associated with amplified linear fragments of chromosome XII rather than ribosomal DNA circle accumulation
Source: PLoS Biol. 2023 Aug 29;21(8):e3002250. doi: 10.1371/journal.pbio.3002250 (PMC10464983; doi:10.1371/journal.pbio.3002250)
Supplement: Attachment — Submitted filename: Zylstra et al response second revision_AZ.docx [file pbio.3002250.s024.docx]

**Zylstra *et al*. response to editors’ and reviewers’ comments**

We thank the reviewers and editors for their time and thoughtful comments.

From the editors:

The team was discussing your Title; it's mostly clear, but I'm not sure that "chromosome XII fragments" quite captures it; also it was noticeable that in the Abstract you mention "amplification" but not "fragments." Looking at your Discussion, my understanding is that these are amplified fragments (large and linear) from that region of chr XII, yes? So I wonder if your title should read "...amplified linear fragments of chromosome XII..." and your Abstract should mention that they're linear fragments up to ~1.8 Mb in size?

Those changes do improve clarity, thank you for the suggestions.

Reviewer #2:

 The authors have done an admirable job addressing reviewers concerns in this major revision of the manuscript. They took the concerns of the reviewers seriously and did their best job even if all concerns cannot be addressed. The message of the manuscript is quite interesting as it attempts to overturn a long held model in the field of yeast aging regarding causality of ERCs. I am still not convinced about the broad relevance to aging biology generally but that is only a minor detraction.

Because the revision is quite extensive I think it is natural to have a few new comments.

The authors have added a figure that contains a model for how the chromosome fragment impacts NPCs to create age related dysfunction. Several reviewers suggested this was needed. The model relies heavily on a previous publication. I'm not clear on why the fragment is predicted to attach to the NPC and create problems whereas normal chromosomes do not. Please clarify in the text.

To our knowledge it is unknown why ERCs are retained in mother cells through binding to NPCs whereas the full chromosome XII is not, and we suspect the difference is chromosome condensation, which in yeast is driven from the centromere. As neither ERCs nor ChrXIIr possess a centromere we would expect both to remain attached to NPC; this remains a hypothesis for now which we have included in the following text:

"It remains unknown why ERCs bind to nuclear pore complexes whereas chromosomal rDNA repeats seemingly do not, though one possibility is that this interaction occurs for all rDNA copies but is disrupted by chromosome condensation which requires a centromere (Kruitwagen et al., 2018). As ERCs and ChrXIIr lack centromeres, both species would remain bound to nuclear pore complexes into mitosis whereas full length chromosome XII would not; in support of this idea, condensin mutants show a specific retention of chromosome XII in the mother cell along with ERCs (Freeman et al., 2000)."

The authors did not remove the original model figure regarding how the fragment forms in the first place. As stated previously, their data does not go very far to demonstrate the mechanism of formation so I was a bit surprised to see it still included although I imagine they have devoted substantial thought regarding the mechanism of formation. It makes for a somewhat unusual format with the conclusion being two model figures explaining two separate processes at the end of the manuscript. Although I do not think its essential to modify the manuscript, I would encourage the authors to consider whether they could either 1) move the mechanism of formation model to the supplement, or 2) combine the two models into a single integrated model that includes both formation and pathology.

Yes, on reflection the reviewer is correct. Putting the two models together proved too complicated, and given that the manuscript focuses on the effects of ChrXIIr rather than the formation it makes sense to move the formation figure to SI (now Figure S10).

Reviewer #3:

The authors have made a big effort to address my concerns and have added a lot of additional data to their study, especially regarding the role of slow growth and cell cycle arrest to SEP associated gene expression. I also appreciate that the authors note in the text that the gene expression data of rad52∆ mutants are outliers and in an effort to remedy these concerns they have added data for additional mutants to the manuscripts, even though some of these mutants suffer from the same issues as rad52 (involvement in DNA damage repair).

While I appreciate the authors effort, I still have some major concerns about the authors interpretation of the results. In particular, I don't see how their data supports two key conclusions:

1)       	The authors refute a role of ERCs in inducing cell senescence, based on the fact that mutants that do not form ERCs can still senesce. I agree with the authors interpretation that ERCs are not necessary for the SEP associated phenotypes (ERCs are not necessary). In fact, cells with irreparable DNA damage (rad52∆) might enter a terminal cell cycle arrest that will have a very similar molecular signature as cell senescence. The fact that cells can senesce without ERCs does not mean that ERCs do not contribute to SEP in wt cells. The plasmid accumulation experiment in Meinema et al 2022 suggest that indeed extrachromosomal plasmids are sufficient. The wording should be adjusted correctly.

What is missing here is the corroboration from the Horkai *et al*. manuscript, in which we show that ERCs are not sufficient to induce SEP phenotypes as cells aged for 48 hours on galactose have similar ERC levels to those aged on glucose but minimal SEP phenotypes. The same point is made more generally by Figure 6G of this manuscript. Therefore our data shows that ERCs are neither necessary nor sufficient to induce SEP phenotypes, which strongly indicates that ERCs are not primary drivers of the SEP in wildtype cells. To make this explicit we have modified the first paragraph of the Discussion as follows:

"Here we examined ageing phenotypes in a panel of low-ERC mutants and found that age-associated transcriptomic dysregulation and the SEP occur independently of ERCs. Furthermore, we did not find any robust indication of pathway specific impacts of ERCs at the gene expression level. ERCs are therefore not necessary for age-associated phenotypes in yeast, and in the accompanying manuscript we show that ERCs are also not sufficient as cells aged for 48 hours on galactose have plentiful ERCs but minimal age-associated phenotypes (Horkai et al.). These findings are incompatible with the hypothesis that ERCs are a primary driver of age-related changes in S. cerevisiae, and instead we show that another rDNA-derived extrachromosomal species is tightly associated with the SEP and may be a major contributor to age-related phenotypes in normal yeast cells."

We addressed the Meinema *et al* study in the Discussion and noted that ERCs may have some contribution and potential causal role, but under the model we propose this effect would be much smaller than the effect of ChrXIIr (in keeping with our observations). This is the relevant statement:

" Others have shown that synthetic ERCs can cause lifespan and nuclear pore defects, although rDNA sequences themselves are dispensable (Falcon and Aris, 2003; Meinema et al., 2022; Sinclair and Guarente, 1997); it is likely that small circular DNAs the size of a single ERC could entangle a few nuclear pores, causing a pathogenic phenotype if  present at sufficient levels, but the model we propose predicts that these would be far less disruptive than a much smaller number of very large linear molecules such as ChrXIIr."

2)       	The authors imply that amplification of XIIr causes SEP, but the data show a correlation, which does not prove causality. It is also possible that the partial aneuploidy is a consequence of impaired DNA damage repair and fatal cell cycle errors that are frequently observed long after cells have started to senesce (see Crane et al 2019 and Neurohr et al 2018). This interpretation fits well with the observation that rad52∆ mutants have increased levels of XIIr accumulation. The fact that XIIr does not accumulate to more than one copy per cell (which the authors suggest but this has not been analyzed) suggests that the translocation happens in the last division and not several divisions before cells stop dividing. In order to make the claim that XIIr contributes to the SEP transition (onset of slow cell divisions, which happens several divisions before cell death), the authors would at least need to show that the XIIr accumulation happens before or at the onset of the SEP, for example by using life span imaging with cells containing TetO repeats on the distal arm of chromosome XII. To establish causality, they would also need to show that preventing the translocation prevents SEP formation. As these would require quite substantial additional experiments, I suggest the authors tone down their language in regard to this model and present it as a possibility while pointing out that this is only one of several possible other interpretations.

We agree that we have not yet proven causality for ChrXIIr. We have been very careful in our wording not to make this claim and instead highlight the strong correlation which we use as the basis to propose a mechanism. The reviewer is also absolutely correct that the copy number of ChrXIIr is low and the correlation of timing between ChrXIIr formation and the SEP needs to be tested at a single cell level. We have made the following changes to make it clear that the model is our interpretation but others are possible, and to highlight the low copy number of ChrXIIr:

Last paragraph of Introduction: “Instead we show a strong correlation between amplification of a large linear fragment of chromosome XII (ChrXIIr) and the SEP, suggesting that this may be the unanticipated cause of senescence in yeast.”

First paragraph of Discussion: "instead we show that another rDNA-derived extrachromosomal species is tightly associated with the SEP and may be a major contributor to age-related phenotypes in normal yeast cells."

Second paragraph of Discussion: "Substantial effort has been invested in understanding the mechanism by which ERC accumulation can cause ageing. Given that ChrXIIr levels are much more tightly associated with the SEP than ERC levels, we asked whether these mechanisms could be adapted to explain how ChrXIIr present at ~1 molecule per cell might induce age-linked phenotypes."

Third  paragraph of Discussion: "it is likely that small circular DNAs the size of a single ERC could entangle a few nuclear pores, causing a pathogenic phenotype if  present at sufficient levels, but the model we propose predicts that these would be far less disruptive than a much smaller number of very large linear molecules such as ChrXIIr."

Sixth paragraph of Discussion: "Our findings suggest that other, lower copy number extrachromosomal DNA species could be potent mediators of ageing phenotypes, driving differences in ageing health almost independent of lifespan. Of course, it is not yet proven that the appearance of ChrXIIr always presages SEP onset so we cannot rule out the possibility that ChrXIIr formation is a consequence rather than a cause of the SEP.  However, non-centromeric fragments have the potential to mediate long term phenotypic effects of ageing and could arise through age-linked rDNA cleavage in higher eukaryotes, raising the prospect of an rDNA stability mechanism of ageing pathology conserved from yeast to mammals."

Minor points:

3)       	By visual inspection it looks like the SEP associated correlation is mostly driven by the similarity between wild type and rad52∆ and the most important mutant spt3∆ (which is not short lived, has no ERCs but enters SEP) shows a much weaker correlation. Rather than comparing very different mutants and growth conditions it would be interesting to see the comparison of only wt and spt3 mutants, which have the most similar trajectories but differ in ERC levels.

The reviewer raises an important point about the *rad52*∆ mutant regarding the correlation between the SEP phenotype and ChrXIIr accumulation. The *rad52*∆ mutant shows essentially no ERCs, a SEP phenotype matching or exceeding the wild-type, and a severe DNA repair deficit. As such, it is a) the mutant which most clearly runs counter to the previously reported ERCs/SEP correlation, and b) a prime candidate for displaying chromosomal abnormalities arising from failed double strand break repair, of which ChrXIIr is likely an example. Based on this we agree that the *rad52*∆ data could plausibly influence our conclusions disproportionately.

Re-plotting some of the figures to compare wild-type and *spt3*∆ would not really help as the choice of significant genes included in each comparison is driven by the linear models, and if any bias arises due to *rad52*∆ it would drive the set of significant genes called by the linear models. Alternatively, we could use linear modelling to compare only the wild-type and *spt3*∆ mutant RNA-seq data. However, fitting multi-factor models to smaller datasets compromises statistical power and worsens problems with correlated independent variables (particularly Age and SEP which tend to correlate). This is why including data from multiple mutants with different ageing characteristics is so valuable – it helps to de-correlate these factors.

Therefore, to address the reviewer’s valid concern regarding Rad52, we have re-fitted linear models 2 and 3 without including the *rad52*∆ data (so only using wild type, *spt3*∆ and *sac3*∆ data) to assess whether the outcomes are substantially altered. Reassuringly, the exclusion of the *rad52*∆ dataset has little effect and our findings are unaffected, as described in the additional text below and additional supplemental figure S8:

" Finally, the particularly strong SEP phenotype of the rad52Δ mutant, as well as its profound DNA repair deficit, could result in the rad52Δ dataset having an undue influence in our linear models wherein we find an association between the SEP and the ChrXIIr chromosomal abnormality. To address this concern, we re-fitted linear models 2 and 3 including data for wild type, spt3Δ and sac3Δ but without the rad52Δ dataset (Supplementary Files 4,5). Reassuringly, most genes significantly differentially expressed based on the SEP variable in the original models were still significantly differentially expressed when the rad52Δ data was excluded (78% of genes in Model 2 and 90% of genes in Model 3). Furthermore, genes significantly differentially expressed based on the SEP were still strongly over-represented on ChrXIIr but not on other chromosomes when rad52Δ was excluded from the analysis, showing that the association of ChrXIIr with the SEP is maintained (Figure S8A). Quantitatively, individual coefficients from model 3 fitted without the rad52Δ dataset were very similar to the original model (median difference in Age coefficient 0.02 ± 0.03 SD, SEP coefficient 0.04 ± 0.09 SD), and unsurprisingly plots of these components are almost identical to those from the model fitted with the full dataset (compare Figure S8B to 4F). Although the component of IGS transcription attributed to the SEP by the model lacking the rad52Δ dataset is higher than in the original model, this is still minor compared to the component associated with Age; this means that ERC amplification is still primarily associated with age rather than SEP onset (Figure S8B red dots). These tests clearly demonstrate that our findings are not dependent on the dataset from the rad52Δ mutant."

4)       	The XIIr <-> V swap experiment is elegant, yet cells still senesce. This could be caused by accumulation of a large rDNA containing fragment as the authors argue, but it is not clear that this translocation still occurs as it is hard/impossible to distinguish from ERC accumulation. Is the control wt strain also an a/a diploid? The reason why this is important is that DNA damage repair pathways are regulated differently in haploid (a/a will behave as a haploid) and diploid (a/alpha) cells. (NHEJ is suppressed in diploids (a/alpha).

Yes, the control strain is also an a/a diploid that exactly matches the translocation strain, made by mating together the haploids in which the translocation strains were generated. We have highlighted this.

Reviewer #4:

 The authors have addressed most of my comments. Regarding my last point (#4), I disagree with them: they could have used the TEF1 promoter or the GAL1 promoter (constitutively expressed in gal80 deletion background) driving stable GFP, as strong promoters; they could have seen expression level differences on the order of 50% using these systems. Average reporter expression levels for similar-age cells across the population can be surprisingly stable across the healthy aging period (Sarnoski et al, 2018, cited already).

We thank the reviewer for this suggestion and will try this approach as we move forward with this work.

Overall, I believe the results from the manuscript will be of interest to the broad readership of PlosBiology and I warmly recommend its acceptance for publication.
